# Supplementary material for: Contribution of systemic and somatic factors to clinical response and resistance to PD-L1 blockade in urothelial cancer: An exploratory multi-omic analysis
Source: PLoS Med. 2017 May 26;14(5):e1002309. doi: 10.1371/journal.pmed.1002309 (PMC5446110; doi:10.1371/journal.pmed.1002309)
Supplement: S1 Table — We chose to optimize for precision, choosing the highlighted set of filters/BQSR. Precision is defined as the fraction of filtered missense mutations in Integrated Mutation Profiling for Actionable Cancer Targets (IMPACT) genes that were actual IMPACT panel variants. Recall is defined as the fraction of actual IMPACT panel variants that were found in the filtered missense results. (DOCX) [file pmed.1002309.s001.docx]

# S1 Table

| DCB AUC | p-value | Filters | With BQSR? | IMPACT Precision | IMPACT Recall |
| --- | --- | --- | --- | --- | --- |
| [0.65, 95% CI (0.43, 0.85)](https://github.com/hammerlab/bladder-analyses/blob/master/analyses/notebooks/Different%20Filters%20vs.%20Benefit.ipynb?hyper=with_bqsr_rizvi_filter_auc) | [n=25, Mann-Whitney p=0.22](https://github.com/hammerlab/bladder-analyses/blob/master/analyses/notebooks/Different%20Filters%20vs.%20Benefit.ipynb?hyper=with_bqsr_rizvi_filter_mw) | [Tumor/Normal Depth ≥ 7, Tumor VAF > 0.1, Normal VAF < 0.03](https://github.com/hammerlab/bladder-analyses/blob/master/analyses/notebooks/Different%20Filters%20vs.%20Benefit.ipynb?hyper=with_bqsr_rizvi_filter_auc_description) | Yes | [0.72](https://github.com/hammerlab/bladder-analyses/blob/master/analyses/notebooks/Different%20Filters%20vs.%20Benefit.ipynb?hyper=with_bqsr_rizvi_filter_precision) | [0.49](https://github.com/hammerlab/bladder-analyses/blob/master/analyses/notebooks/Different%20Filters%20vs.%20Benefit.ipynb?hyper=with_bqsr_rizvi_filter_recall) |
| [0.61, 95% CI (0.35, 0.85)](https://github.com/hammerlab/bladder-analyses/blob/master/analyses/notebooks/Different%20Filters%20vs.%20Benefit.ipynb?hyper=with_bqsr_filter_30_auc) | [n=25, Mann-Whitney p=0.41](https://github.com/hammerlab/bladder-analyses/blob/master/analyses/notebooks/Different%20Filters%20vs.%20Benefit.ipynb?hyper=with_bqsr_filter_30_mw) | [Tumor/Normal Depth ≥ 30, Tumor Alt Depth ≥ 5](https://github.com/hammerlab/bladder-analyses/blob/master/analyses/notebooks/Different%20Filters%20vs.%20Benefit.ipynb?hyper=with_bqsr_filter_30_auc_description) | Yes | [0.68](https://github.com/hammerlab/bladder-analyses/blob/master/analyses/notebooks/Different%20Filters%20vs.%20Benefit.ipynb?hyper=with_bqsr_filter_30_precision) | [0.52](https://github.com/hammerlab/bladder-analyses/blob/master/analyses/notebooks/Different%20Filters%20vs.%20Benefit.ipynb?hyper=with_bqsr_filter_30_recall) |
| [0.63, 95% CI (0.36, 0.86)](https://github.com/hammerlab/bladder-analyses/blob/master/analyses/notebooks/Different%20Filters%20vs.%20Benefit.ipynb?hyper=with_bqsr_no_filter_auc) | [n=25, Mann-Whitney p=0.29](https://github.com/hammerlab/bladder-analyses/blob/master/analyses/notebooks/Different%20Filters%20vs.%20Benefit.ipynb?hyper=with_bqsr_no_filter_mw) | [No Depth/VAF Filters](https://github.com/hammerlab/bladder-analyses/blob/master/analyses/notebooks/Different%20Filters%20vs.%20Benefit.ipynb?hyper=with_bqsr_no_filter_auc_description) | Yes | [0.68](https://github.com/hammerlab/bladder-analyses/blob/master/analyses/notebooks/Different%20Filters%20vs.%20Benefit.ipynb?hyper=with_bqsr_no_filter_precision) | [0.63](https://github.com/hammerlab/bladder-analyses/blob/master/analyses/notebooks/Different%20Filters%20vs.%20Benefit.ipynb?hyper=with_bqsr_no_filter_recall) |
| [0.72, 95% CI (0.51, 0.90)](https://github.com/hammerlab/bladder-analyses/blob/master/analyses/notebooks/Different%20Filters%20vs.%20Benefit.ipynb?hyper=without_bqsr_rizvi_filter_auc) | [n=25, Mann-Whitney p=0.075](https://github.com/hammerlab/bladder-analyses/blob/master/analyses/notebooks/Different%20Filters%20vs.%20Benefit.ipynb?hyper=without_bqsr_rizvi_filter_mw) | [Tumor/Normal Depth ≥ 7, Tumor VAF > 0.1, Normal VAF < 0.03](https://github.com/hammerlab/bladder-analyses/blob/master/analyses/notebooks/Different%20Filters%20vs.%20Benefit.ipynb?hyper=without_bqsr_rizvi_filter_auc_description) | No | [0.63](https://github.com/hammerlab/bladder-analyses/blob/master/analyses/notebooks/Different%20Filters%20vs.%20Benefit.ipynb?hyper=without_bqsr_rizvi_filter_precision) | [0.5](https://github.com/hammerlab/bladder-analyses/blob/master/analyses/notebooks/Different%20Filters%20vs.%20Benefit.ipynb?hyper=without_bqsr_rizvi_filter_recall) |
| [0.63, 95% CI (0.38, 0.86)](https://github.com/hammerlab/bladder-analyses/blob/master/analyses/notebooks/Different%20Filters%20vs.%20Benefit.ipynb?hyper=without_bqsr_filter_30_auc) | [n=25, Mann-Whitney p=0.29](https://github.com/hammerlab/bladder-analyses/blob/master/analyses/notebooks/Different%20Filters%20vs.%20Benefit.ipynb?hyper=without_bqsr_filter_30_mw) | [Tumor/Normal Depth ≥ 30, Tumor Alt Depth ≥ 5](https://github.com/hammerlab/bladder-analyses/blob/master/analyses/notebooks/Different%20Filters%20vs.%20Benefit.ipynb?hyper=without_bqsr_filter_30_auc_description) | No | [0.68](https://github.com/hammerlab/bladder-analyses/blob/master/analyses/notebooks/Different%20Filters%20vs.%20Benefit.ipynb?hyper=without_bqsr_filter_30_precision) | [0.52](https://github.com/hammerlab/bladder-analyses/blob/master/analyses/notebooks/Different%20Filters%20vs.%20Benefit.ipynb?hyper=without_bqsr_filter_30_recall) |
| [0.72, 95% CI (0.48, 0.90)](https://github.com/hammerlab/bladder-analyses/blob/master/analyses/notebooks/Different%20Filters%20vs.%20Benefit.ipynb?hyper=without_bqsr_no_filter_auc) | [n=25, Mann-Whitney p=0.084](https://github.com/hammerlab/bladder-analyses/blob/master/analyses/notebooks/Different%20Filters%20vs.%20Benefit.ipynb?hyper=without_bqsr_no_filter_mw) | [No Depth/VAF Filters](https://github.com/hammerlab/bladder-analyses/blob/master/analyses/notebooks/Different%20Filters%20vs.%20Benefit.ipynb?hyper=without_bqsr_no_filter_auc_description) | No | [0.53](https://github.com/hammerlab/bladder-analyses/blob/master/analyses/notebooks/Different%20Filters%20vs.%20Benefit.ipynb?hyper=without_bqsr_no_filter_precision) | [0.65](https://github.com/hammerlab/bladder-analyses/blob/master/analyses/notebooks/Different%20Filters%20vs.%20Benefit.ipynb?hyper=without_bqsr_no_filter_recall) |

Choice of depth and VAF filtering, as well as whether or not to run Base Quality Score Recalibration (BQSR), resulted in differences in predictive value for mutation load. We chose to optimize for precision, choosing the highlighted set of filters/BQSR. Precision is defined as the fraction of filtered missense mutations in IMPACT genes that were actual IMPACT panel variants. Recall is defined as the fraction of actual IMPACT panel variants that were found in the filtered missense results.
