# Supplementary material for: Contribution of systemic and somatic factors to clinical response and resistance to PD-L1 blockade in urothelial cancer: An exploratory multi-omic analysis
Source: PLoS Med. 2017 May 26;14(5):e1002309. doi: 10.1371/journal.pmed.1002309 (PMC5446110; doi:10.1371/journal.pmed.1002309)
Supplement: S2 Table — Results are summarized as median and 95% posterior intervals. (DOCX) [file pmed.1002309.s002.docx]

# S2 Table

| Metric | PD-L1 Expression (IC group) | HR for Progression or Mortality | HR for Mortality |
| --- | --- | --- | --- |
| Intercept | | | |
|  | IC0 | (base group) | (base group) |
|  | IC1 | [HR=0.29, 95% CI (0.079, 1.36)](https://github.com/hammerlab/bladder-analyses/blob/master/analyses/notebooks/Multivariate%20models.ipynb?hyper=m0pfs_intercept_IC1) | [HR=0.23, 95% CI (0.054, 1.10)](https://github.com/hammerlab/bladder-analyses/blob/master/analyses/notebooks/Multivariate%20models.ipynb?hyper=m0os_intercept_IC1) |
|  | IC2 | [HR=0.083, 95% CI (0.0087, 0.55)](https://github.com/hammerlab/bladder-analyses/blob/master/analyses/notebooks/Multivariate%20models.ipynb?hyper=m0pfs_intercept_IC2) | [HR=0.023, 95% CI (0.00047, 0.30)](https://github.com/hammerlab/bladder-analyses/blob/master/analyses/notebooks/Multivariate%20models.ipynb?hyper=m0os_intercept_IC2) |
| Liver Metastasis | | | |
|  | IC0 | [HR=4.17, 95% CI (0.53, 33.98)](https://github.com/hammerlab/bladder-analyses/blob/master/analyses/notebooks/Multivariate%20models.ipynb?hyper=m0pfs_liver_mets_IC0) | [HR=4.78, 95% CI (0.58, 53.10)](https://github.com/hammerlab/bladder-analyses/blob/master/analyses/notebooks/Multivariate%20models.ipynb?hyper=m0os_liver_mets_IC0) |
|  | IC1 | [HR=4.27, 95% CI (1.15, 15.08)](https://github.com/hammerlab/bladder-analyses/blob/master/analyses/notebooks/Multivariate%20models.ipynb?hyper=m0pfs_liver_mets_IC1) | [HR=4.11, 95% CI (0.91, 16.01)](https://github.com/hammerlab/bladder-analyses/blob/master/analyses/notebooks/Multivariate%20models.ipynb?hyper=m0os_liver_mets_IC1) |
|  | IC2 | [HR=3.80, 95% CI (0.79, 22.70)](https://github.com/hammerlab/bladder-analyses/blob/master/analyses/notebooks/Multivariate%20models.ipynb?hyper=m0pfs_liver_mets_IC2) | [HR=3.73, 95% CI (0.61, 24.52)](https://github.com/hammerlab/bladder-analyses/blob/master/analyses/notebooks/Multivariate%20models.ipynb?hyper=m0os_liver_mets_IC2) |
| log(# Missense SNV / MB) | | | |
|  | IC0 | [HR=1.00, 95% CI (0.49, 2.37)](https://github.com/hammerlab/bladder-analyses/blob/master/analyses/notebooks/Multivariate%20models.ipynb?hyper=m0pfs_log_missense_snv_count_centered_by_pd_l1_IC0) | [HR=1.26, 95% CI (0.60, 3.19)](https://github.com/hammerlab/bladder-analyses/blob/master/analyses/notebooks/Multivariate%20models.ipynb?hyper=m0os_log_missense_snv_count_centered_by_pd_l1_IC0) |
|  | IC1 | [HR=0.76, 95% CI (0.50, 1.16)](https://github.com/hammerlab/bladder-analyses/blob/master/analyses/notebooks/Multivariate%20models.ipynb?hyper=m0pfs_log_missense_snv_count_centered_by_pd_l1_IC1) | [HR=0.83, 95% CI (0.50, 1.28)](https://github.com/hammerlab/bladder-analyses/blob/master/analyses/notebooks/Multivariate%20models.ipynb?hyper=m0os_log_missense_snv_count_centered_by_pd_l1_IC1) |
|  | IC2 | [HR=0.53, 95% CI (0.33, 0.83)](https://github.com/hammerlab/bladder-analyses/blob/master/analyses/notebooks/Multivariate%20models.ipynb?hyper=m0pfs_log_missense_snv_count_centered_by_pd_l1_IC2) | [HR=0.56, 95% CI (0.33, 1.00)](https://github.com/hammerlab/bladder-analyses/blob/master/analyses/notebooks/Multivariate%20models.ipynb?hyper=m0os_log_missense_snv_count_centered_by_pd_l1_IC2) |
| log(Pre-Treatment TCR Clonality) | | | |
|  | IC0 | [HR=1.51, 95% CI (0.32, 7.22)](https://github.com/hammerlab/bladder-analyses/blob/master/analyses/notebooks/Multivariate%20models.ipynb?hyper=m0pfs_log_peripheral_clonality_a_centered_by_pd_l1_IC0) | [HR=1.08, 95% CI (0.21, 4.15)](https://github.com/hammerlab/bladder-analyses/blob/master/analyses/notebooks/Multivariate%20models.ipynb?hyper=m0os_log_peripheral_clonality_a_centered_by_pd_l1_IC0) |
|  | IC1 | [HR=1.09, 95% CI (0.32, 3.46)](https://github.com/hammerlab/bladder-analyses/blob/master/analyses/notebooks/Multivariate%20models.ipynb?hyper=m0pfs_log_peripheral_clonality_a_centered_by_pd_l1_IC1) | [HR=1.09, 95% CI (0.33, 3.81)](https://github.com/hammerlab/bladder-analyses/blob/master/analyses/notebooks/Multivariate%20models.ipynb?hyper=m0os_log_peripheral_clonality_a_centered_by_pd_l1_IC1) |
|  | IC2 | [HR=50.87, 95% CI (2.99, 1486.55)](https://github.com/hammerlab/bladder-analyses/blob/master/analyses/notebooks/Multivariate%20models.ipynb?hyper=m0pfs_log_peripheral_clonality_a_centered_by_pd_l1_IC2) | [HR=4895.08, 95% CI (80.81, 10374151.41)](https://github.com/hammerlab/bladder-analyses/blob/master/analyses/notebooks/Multivariate%20models.ipynb?hyper=m0os_log_peripheral_clonality_a_centered_by_pd_l1_IC2) |
| log(TIL Proportion) | | | |
|  | IC0 | [HR=0.12, 95% CI (0.0028, 3.46)](https://github.com/hammerlab/bladder-analyses/blob/master/analyses/notebooks/Multivariate%20models.ipynb?hyper=m0pfs_log_tcell_fraction_centered_by_pd_l1_IC0) | [HR=0.053, 95% CI (0.00053, 11.87)](https://github.com/hammerlab/bladder-analyses/blob/master/analyses/notebooks/Multivariate%20models.ipynb?hyper=m0os_log_tcell_fraction_centered_by_pd_l1_IC0) |
|  | IC1 | [HR=0.11, 95% CI (0.0077, 1.20)](https://github.com/hammerlab/bladder-analyses/blob/master/analyses/notebooks/Multivariate%20models.ipynb?hyper=m0pfs_log_tcell_fraction_centered_by_pd_l1_IC1) | [HR=0.047, 95% CI (0.0018, 0.68)](https://github.com/hammerlab/bladder-analyses/blob/master/analyses/notebooks/Multivariate%20models.ipynb?hyper=m0os_log_tcell_fraction_centered_by_pd_l1_IC1) |
|  | IC2 | [HR=0.078, 95% CI (0.0051, 1.07)](https://github.com/hammerlab/bladder-analyses/blob/master/analyses/notebooks/Multivariate%20models.ipynb?hyper=m0pfs_log_tcell_fraction_centered_by_pd_l1_IC2) | [HR=0.026, 95% CI (0.00093, 0.40)](https://github.com/hammerlab/bladder-analyses/blob/master/analyses/notebooks/Multivariate%20models.ipynb?hyper=m0os_log_tcell_fraction_centered_by_pd_l1_IC2) |

Summary of results from multivariate survival analysis of various clinical, peripheral and intratumoral factors to estimate their independent association with hazard for disease progression or mortality (PFS) and for mortality (OS) according to level of intratumoral PD-L1 expression (IC grade). Results are summarized as median and 95% posterior intervals.
