# Supplementary material for: Contribution of systemic and somatic factors to clinical response and resistance to PD-L1 blockade in urothelial cancer: An exploratory multi-omic analysis
Source: PLoS Med. 2017 May 26;14(5):e1002309. doi: 10.1371/journal.pmed.1002309 (PMC5446110; doi:10.1371/journal.pmed.1002309)
Supplement: S1 Fig — (A) Twenty-five percent of patients with durable clinical benefit (DCB) had less than the median tumor-infiltrating T lymphocytes (TIL) proportion versus 63% of patients without DCB (n = 24, Fisher's Exact p = 0.19); similarly, 25% of patients with DCB had less than the median TIL clonality versus 63% of patients without DCB (n = 24, Fisher's Exact p = 0.19). (B) T cell receptor (TCR) overlap between the pretreatment and 3-week posttreatment peripheral blood in 1 patient with limited clinical benefit (progression-free survival [PFS] = 37 days) and 1 patient with DCB (complete response [CR] at 630 days after starting treatment). The association between pretreatment peripheral blood TCR sequences (x-axis) and posttreatment peripheral blood TCR sequences (y-axis) is overlaid with the presence of tumor-associated T cell clones. Gray indicates TCRs present only in the peripheral blood; blue indicates TCRs present in the tumor and blood; orange indicates TCRs present in the tumor and expanded in the blood with treatment. (C) There was no significant expansion of TIL-associated TCR clones between pretreatment (3.00 [range 1.00–9.00]) and 6 weeks posttreatment (2.00 [range 1.00–8.00]), n = 20, Mann-Whitney p = 0.17. (D) The combination of high pretreatment TIL and low pretreatment peripheral blood TCR clonality were predictive of DCB (n = 24, Fisher's Exact p = 0.0069) and overall survival greater than 12 months (DCB-OS) (n = 24, Fisher's Exact p = 0.014). For DCB, a logit model combining both was more predictive than peripheral blood (n = 24, log-likelihood p = 0.00029) or TIL (n = 24, log-likelihood p = 0.00051) clonality alone. For DCB-OS, both combined were more predictive than TIL (n = 24, log-likelihood p = 0.0029) clonality alone. (DOCX) [file pmed.1002309.s003.docx]

# S1 Fig

## S1A Fig


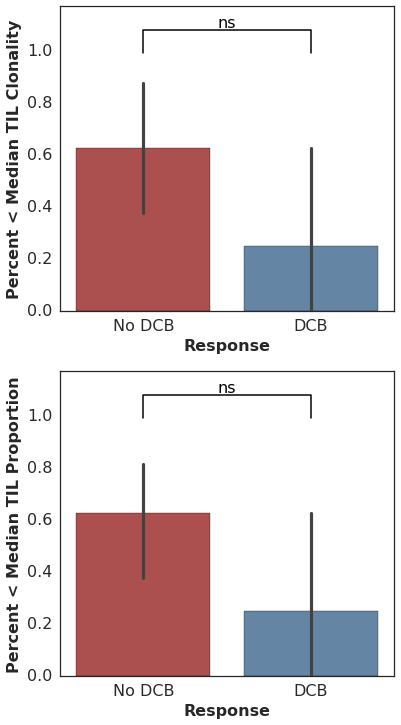


[25%](https://github.com/hammerlab/bladder-analyses/blob/master/analyses/notebooks/T-cell%20Fraction%20vs.%20Clonality%20Updated.ipynb?hyper=below_median_til_fraction_dcb_benefit) of patients with DCB had less than the median TIL proportion versus [63%](https://github.com/hammerlab/bladder-analyses/blob/master/analyses/notebooks/T-cell%20Fraction%20vs.%20Clonality%20Updated.ipynb?hyper=below_median_til_fraction_dcb_no_benefit) of patients without DCB ([n=24, Fisher's Exact p=0.19](https://github.com/hammerlab/bladder-analyses/blob/master/analyses/notebooks/T-cell%20Fraction%20vs.%20Clonality%20Updated.ipynb?hyper=below_median_til_fraction_dcb_fishers)); similarly, [25%](https://github.com/hammerlab/bladder-analyses/blob/master/analyses/notebooks/T-cell%20Fraction%20vs.%20Clonality%20Updated.ipynb?hyper=below_median_til_clonality_dcb_benefit) of patients with DCB had less than the median TIL clonality versus [63%](https://github.com/hammerlab/bladder-analyses/blob/master/analyses/notebooks/T-cell%20Fraction%20vs.%20Clonality%20Updated.ipynb?hyper=below_median_til_clonality_dcb_no_benefit) of patients without DCB ([n=24, Fisher's Exact p=0.19](https://github.com/hammerlab/bladder-analyses/blob/master/analyses/notebooks/T-cell%20Fraction%20vs.%20Clonality%20Updated.ipynb?hyper=below_median_til_clonality_dcb_fishers)).

## S1B Fig


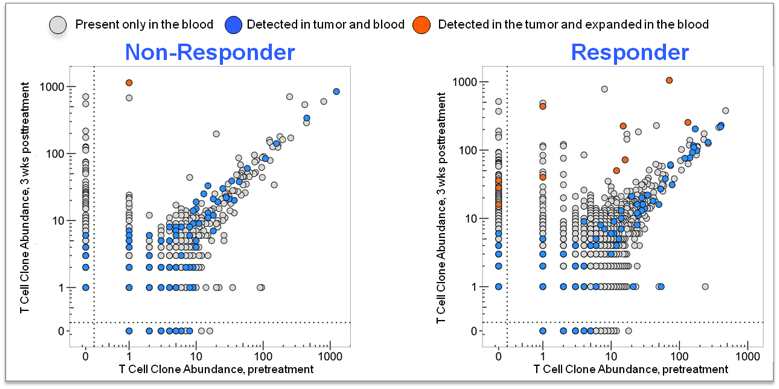


TCR overlap between the pre-treatment and 3-week post-treatment peripheral blood in one patient with limited clinical benefit (PFS=37 days) and one patient with durable clinical benefit (CR at 630 days after starting treatment). The association between pre-treatment peripheral blood TCR sequences (x axis) and post-treatment peripheral blood TCR sequences (y axis) is overlaid with the presence of tumor associated T cell clones. Gray indicates TCRs present only in the peripheral blood; blue indicates TCRs present in the tumor and blood; orange indicates TCRs present in the tumor and expanded in the blood with treatment.

## S1C Fig


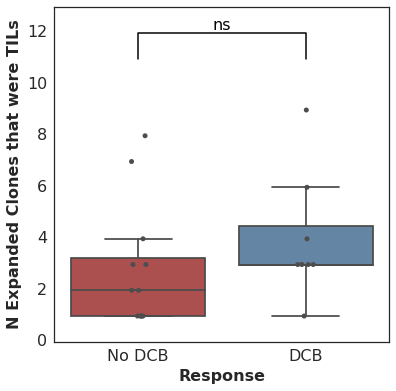


There was no significant expansion of TIL-associated TCR clones between pre-treatment ([3.00 (range 1.00-9.00)](https://github.com/hammerlab/bladder-analyses/blob/master/analyses/notebooks/Adaptive%20Plots.ipynb?hyper=a_c_til_expansion_benefit)) and 6 weeks post-treatment ([2.00 (range 1.00-8.00)](https://github.com/hammerlab/bladder-analyses/blob/master/analyses/notebooks/Adaptive%20Plots.ipynb?hyper=a_c_til_expansion_no_benefit)), [n=20, Mann-Whitney p=0.17](https://github.com/hammerlab/bladder-analyses/blob/master/analyses/notebooks/Adaptive%20Plots.ipynb?hyper=a_c_til_expansion_mw).

## S1D Fig


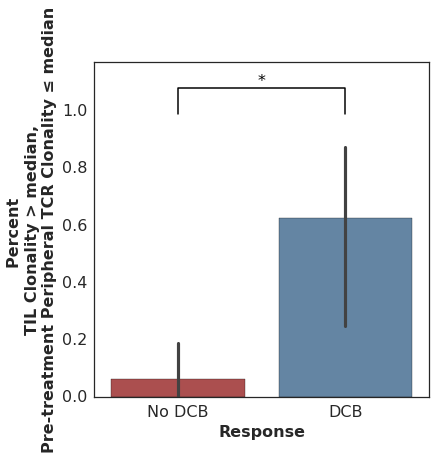


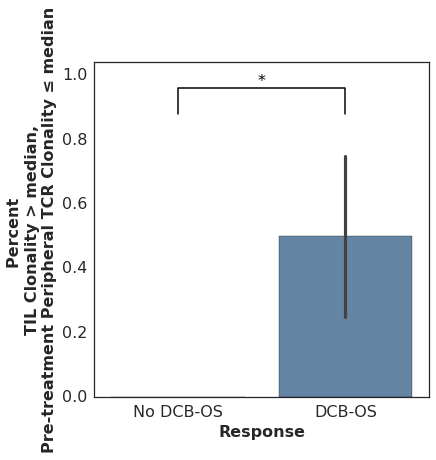


The combination of high pre-treatment TIL and low pre-treatment peripheral blood TCR clonality were predictive of DCB ([n=24, Fisher's Exact p=0.0069](https://github.com/hammerlab/bladder-analyses/blob/master/analyses/notebooks/TIL%20vs.%20Blood%20Clonality.ipynb?hyper=til_high_blood_low_pfs_fishers)) and DCB-OS ([n=24, Fisher's Exact p=0.014](https://github.com/hammerlab/bladder-analyses/blob/master/analyses/notebooks/TIL%20vs.%20Blood%20Clonality.ipynb?hyper=til_high_blood_low_os_fishers)). For DCB, a logit model combining both was more predictive than peripheral blood ([n=24, log-likelihood p=0.00029](https://github.com/hammerlab/bladder-analyses/blob/master/analyses/notebooks/TIL%20vs.%20Blood%20Clonality.ipynb?hyper=llr_blood_tumor_vs_blood_pfs)) or TIL ([n=24, log-likelihood p=0.00051](https://github.com/hammerlab/bladder-analyses/blob/master/analyses/notebooks/TIL%20vs.%20Blood%20Clonality.ipynb?hyper=llr_blood_tumor_vs_tumor_pfs)) clonality alone. For DCB-OS, both combined were more predictive than TIL ([n=24, log-likelihood p=0.0029](https://github.com/hammerlab/bladder-analyses/blob/master/analyses/notebooks/TIL%20vs.%20Blood%20Clonality.ipynb?hyper=llr_blood_tumor_vs_tumor_os)) clonality alone.
