# Supplementary material for: Contribution of systemic and somatic factors to clinical response and resistance to PD-L1 blockade in urothelial cancer: An exploratory multi-omic analysis
Source: PLoS Med. 2017 May 26;14(5):e1002309. doi: 10.1371/journal.pmed.1002309 (PMC5446110; doi:10.1371/journal.pmed.1002309)
Supplement: S2 Fig — (A) No significant association between the number of missense single nucleotide variants (SNVs) per megabase and overall survival greater than 12 months (DCB-OS), with 2.13 (range 0.038–11.46) in tumors from those patients who survived greater than 12 months versus 0.48 (range 0.019–9.90) in those who did not (n = 25, Mann-Whitney p = 0.37). (B) No significant difference was found between median expressed neoantigens in tumors from patients who survived greater than or equal to 12 months: 1.31 (range 0.00–6.06) versus 0.35 (range 0.00–5.30) in those who survived less than 12 months (n = 25, Mann-Whitney p = 0.36). (C) No significant difference was found between median predicted neoantigens per megabase: 4.58 (range 0.037–39.48) in tumors from patients with durable clinical benefit (DCB) as compared to 1.35 (range 0.00–20.22) in those who progressed in less than 6 months (no DCB) (n = 25, Mann-Whitney p = 0.55). (D) No significant difference was found between median predicted neoantigens per megabase in tumors from those patients who survived greater than 12 months (3.56 [range 0.037-39.48]) as compared to 1.37 (range 0.00–20.22) in those who did not (no DCB-OS) (n = 25, Mann-Whitney p = 0.81). (DOCX) [file pmed.1002309.s004.docx]

# S2 Fig

## Fig S2A


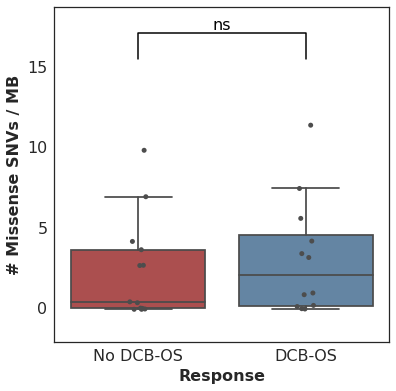


No significant association between the number of missense SNV per megabase and overall survival, with [2.13 (range 0.038-11.46)](https://github.com/hammerlab/bladder-analyses/blob/master/analyses/notebooks/Count%20Plots.ipynb?hyper=os_missense_snv_count_benefit) in tumors from those patients who survived greater than 12 months, versus [0.48 (range 0.019-9.90)](https://github.com/hammerlab/bladder-analyses/blob/master/analyses/notebooks/Count%20Plots.ipynb?hyper=os_missense_snv_count_no_benefit) in those who did not ([n=25, Mann-Whitney p=0.37](https://github.com/hammerlab/bladder-analyses/blob/master/analyses/notebooks/Count%20Plots.ipynb?hyper=os_missense_snv_count_mw)).

## Fig S2B


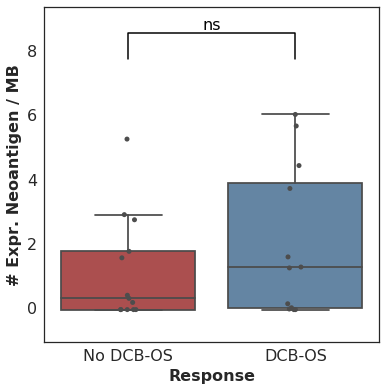


No significant difference between median expressed neoantigens in tumors from patients who survived greater than or equal to 12 months: was [1.31 (range 0.00-6.06)](https://github.com/hammerlab/bladder-analyses/blob/master/analyses/notebooks/Count%20Plots.ipynb?hyper=os_expressed_neoantigen_count_benefit), versus [0.35 (range 0.00-5.30)](https://github.com/hammerlab/bladder-analyses/blob/master/analyses/notebooks/Count%20Plots.ipynb?hyper=os_expressed_neoantigen_count_no_benefit) in those who survived less than 12 months ([n=25, Mann-Whitney p=0.36](https://github.com/hammerlab/bladder-analyses/blob/master/analyses/notebooks/Count%20Plots.ipynb?hyper=os_expressed_neoantigen_count_mw)).

## Fig S2C


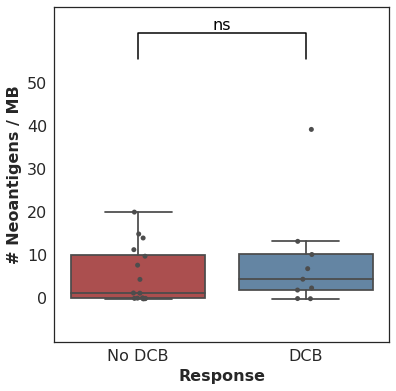


No significant difference between median predicted neoantigens per megabase: [4.58 (range 0.037-39.48)](https://github.com/hammerlab/bladder-analyses/blob/master/analyses/notebooks/Count%20Plots.ipynb?hyper=pfs_neoantigen_count_benefit) in tumors from patients with DCB, as compared to [1.35 (range 0.00-20.22)](https://github.com/hammerlab/bladder-analyses/blob/master/analyses/notebooks/Count%20Plots.ipynb?hyper=pfs_neoantigen_count_no_benefit) in those who progressed in less than 6 months (no DCB) ([n=25, Mann-Whitney p=0.55](https://github.com/hammerlab/bladder-analyses/blob/master/analyses/notebooks/Count%20Plots.ipynb?hyper=pfs_neoantigen_count_mw)).

## Fig S2D


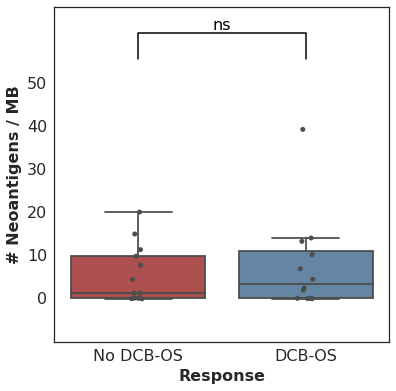


No significant difference between median predicted neoantigens per megabase in tumors from those patients who survived greater than 12 months (here used to define DCB-OS) was [3.56 (range 0.037-39.48)](https://github.com/hammerlab/bladder-analyses/blob/master/analyses/notebooks/Count%20Plots.ipynb?hyper=os_neoantigen_count_benefit) as compared to [1.37 (range 0.00-20.22)](https://github.com/hammerlab/bladder-analyses/blob/master/analyses/notebooks/Count%20Plots.ipynb?hyper=os_neoantigen_count_no_benefit) in those who did not (no DCB) ([n=25, Mann-Whitney p=0.81](https://github.com/hammerlab/bladder-analyses/blob/master/analyses/notebooks/Count%20Plots.ipynb?hyper=os_neoantigen_count_mw)).
