# Supplementary material for: Contribution of systemic and somatic factors to clinical response and resistance to PD-L1 blockade in urothelial cancer: An exploratory multi-omic analysis
Source: PLoS Med. 2017 May 26;14(5):e1002309. doi: 10.1371/journal.pmed.1002309 (PMC5446110; doi:10.1371/journal.pmed.1002309)
Supplement: S3 Fig — (A) Patients who survived less than 3 months (red box) exhibited a significantly higher 5-factor score (3.00 [range 2.00–4.00] as compared to 1.50 [range 0.00–4.00] in patients who survived >3 months; blue box; n = 26, Mann-Whitney p = 0.018). (B) Patients who survived less than or equal to 3 months (red box) were more likely to have liver metastases (100% in patients who survived less than or equal to 3 months and 22% in patients who survived longer than 3 months, n = 29, Fisher's Exact p = 0.00097). (C) The hazard ratio for each mutation per megabase, estimated at each unique failure time. Red box plots summarize 50% and 95% posterior intervals for each observed failure/censor time, with median values shown in green. Time (Days) is plotted on a log-scale. Estimates are not independent from one another since the model utilizes a random-walk parameterization to allow the variance in hazard over time to be modeled flexibly. (D) Posterior predicted intervals for progression-free survival (PFS), which are drawn from the survival model estimating the time-varying effect of mutation count on PFS. Intervals are shown for patients with missense single nucleotide variants (SNVs) per megabase above the median and those with counts below the median value (blue), for illustrative purposes. This cutpoint was not used in the model; missense SNV per megabase was included as a continuous covariate. Lines are drawn at median values of the posterior predictive distribution, with 50% credible intervals shown in the shaded regions. Time (Days) is plotted on a log-scale. (DOCX) [file pmed.1002309.s005.docx]

# S3 Fig

## S3A Fig


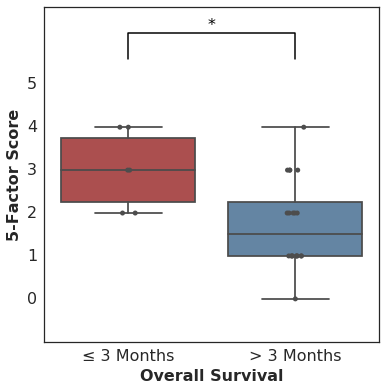


Patients who survived less than 3 months (red box) exhibited a significantly higher 5-factor score ([3.00 (range 2.00-4.00)](https://github.com/hammerlab/bladder-analyses/blob/master/analyses/notebooks/Patients%20with%20%3E3%20months%20survival.ipynb?hyper=late_deceased_five_factor_no_late_deceased), as compared to [1.50 (range 0.00-4.00)](https://github.com/hammerlab/bladder-analyses/blob/master/analyses/notebooks/Patients%20with%20%3E3%20months%20survival.ipynb?hyper=late_deceased_five_factor_late_deceased) in patients who survived >3mo (blue box) ([n=26, Mann-Whitney p=0.018](https://github.com/hammerlab/bladder-analyses/blob/master/analyses/notebooks/Patients%20with%20%3E3%20months%20survival.ipynb?hyper=late_deceased_five_factor_mw)).

## S3B Fig


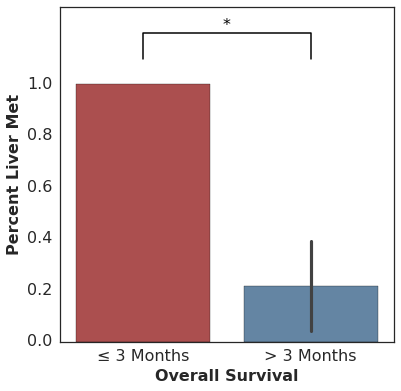


Patients who survived less than or equal to 3 months (red box) were more likely to have liver metastases ([100%](https://github.com/hammerlab/bladder-analyses/blob/master/analyses/notebooks/Patients%20with%20%3E3%20months%20survival.ipynb?hyper=late_deceased_liver_no_late_deceased) in patients who survived less than or equal to 3 months and [22%](https://github.com/hammerlab/bladder-analyses/blob/master/analyses/notebooks/Patients%20with%20%3E3%20months%20survival.ipynb?hyper=late_deceased_liver_late_deceased) in patients who survived longer than 3 months, [n=29, Fisher's Exact p=0.00097](https://github.com/hammerlab/bladder-analyses/blob/master/analyses/notebooks/Patients%20with%20%3E3%20months%20survival.ipynb?hyper=late_deceased_liver_fishers)).

## S3C Fig


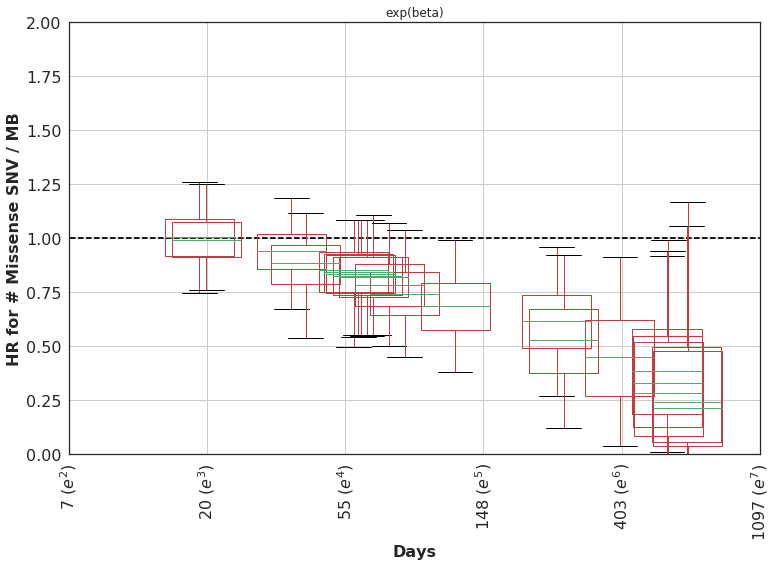


The hazard ratio for each mutation per megabase, estimated at each unique failure time. Red box plots summarize 50% and 95% posterior intervals for each observed failure/censor time, with median values shown in green. Time (Days) is plotted on a log-scale. Estimates are not independent from one another since the model utilizes a random-walk parameterization to allow the variance in hazard over time to be modeled flexibly.

##

## S3D Fig


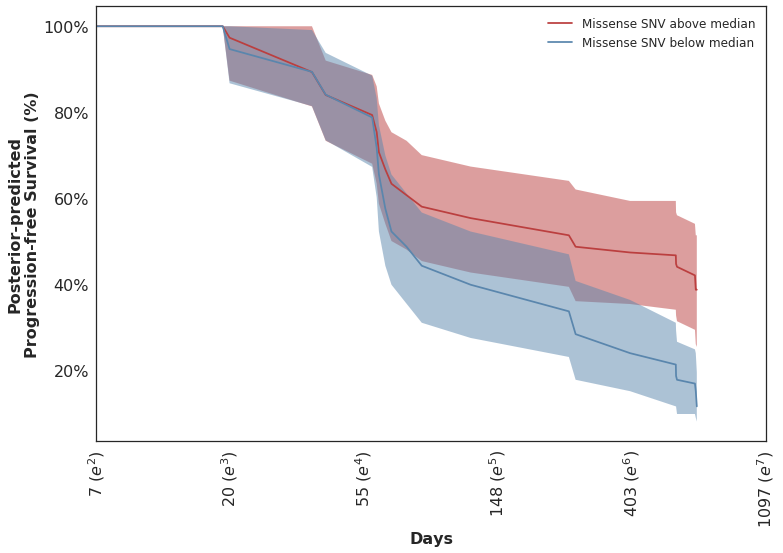


Posterior predicted intervals for PFS drawn from the survival model estimating the time-varying effect of mutation count on PFS. Intervals are shown for patients with missense SNV per megabase above the median and those with counts below the median value (blue) for illustrative purposes. This cutpoint was not used in the model; missense SNV per megabase was included as a continuous covariate. Lines are drawn at median values of the posterior predictive distribution, with 50% credible intervals shown in the shaded regions. Time (Days) is plotted on a log-scale.
