# Supplementary material for: Contribution of systemic and somatic factors to clinical response and resistance to PD-L1 blockade in urothelial cancer: An exploratory multi-omic analysis
Source: PLoS Med. 2017 May 26;14(5):e1002309. doi: 10.1371/journal.pmed.1002309 (PMC5446110; doi:10.1371/journal.pmed.1002309)
Supplement: S4 Fig — (A) Programmed death-ligand 1 (PD-L1) immune cell (IC) staining as reported by the sponsor in the published study [2], and outcomes in our cohort were significantly associated in this substudy (n = 29, Spearman rho = 0.48 p = 0.0083). (B) ImmuneScore was associated with tumor-infiltrating T lymphocytes (TIL) proportion (n = 24, Spearman rho = 0.47 p = 0.022). (C) There was no association between ImmuneScore and durable clinical benefit (DCB) (DCB: 764.37 [range −1195.08 to 1509.65]; no DCB: 263.49 [range −1100.78 to 1734.28]) (n = 26, Mann-Whitney p = 0.33). (D) PD-L1 expression as measured by RNA sequencing was not associated with PD-L1 IC level (n = 26, Spearman rho = 0.045 p = 0.83). Tumor cell PD-L1 staining was not available. (E) Human leukocyte antigen (HLA) Class I expression was not associated with DCB (HLA-A: n = 26, Mann-Whitney p = 0.26, HLA-B: n = 26, Mann-Whitney p = 0.36, HLA-C: n = 26, Mann-Whitney p = 0.24). (F) Expression of other inhibitory markers, in particular HAVCR2 (also known as TIM-3), was higher in DCB patients in the IC2 group. (G) No association was found between TCGA RNA Subtype and response in this substudy (n = 20, Fisher's Exact p = 0.36). (DOCX) [file pmed.1002309.s006.docx]

# S4 Fig

## S4A Fig


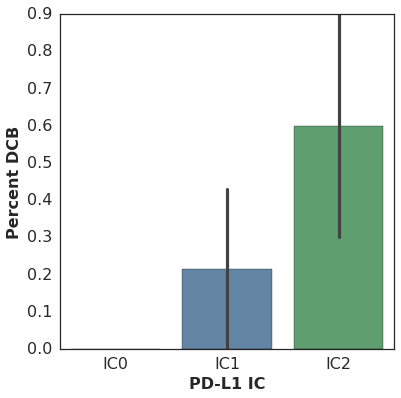


PD-L1 IC staining as reported by the sponsor in the published study [(2)](https://paperpile.com/c/QI3NxB/AkjR) and outcome in our cohort were significantly associated in this sub-study ([n=29, Spearman rho=0.48 p=0.0083](https://github.com/hammerlab/bladder-analyses/blob/master/analyses/notebooks/PD-L1%20vs.%20Benefit.ipynb?hyper=pdl1_any_vs_benefit_spearmanr)).

## S4B Fig


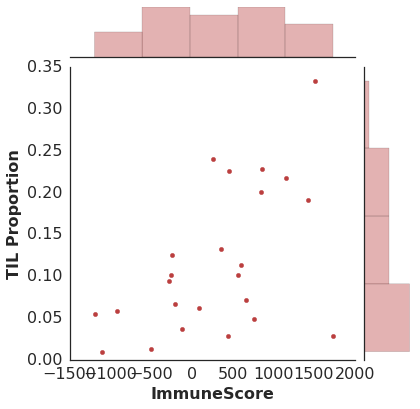


ImmuneScore was associated with TIL proportion ([n=24, Spearman rho=0.47 p=0.022](https://github.com/hammerlab/bladder-analyses/blob/master/analyses/notebooks/ESTIMATE%20immune%20score.ipynb?hyper=estimate_tcellfraction_spearmanr)).

## S4C Fig


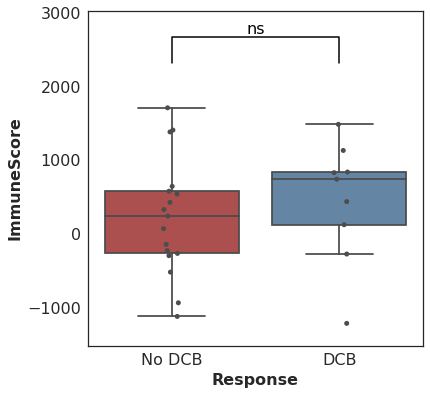


There was no association between ImmuneScore and DCB (DCB, [764.37 (range -1195.08-1509.65)](https://github.com/hammerlab/bladder-analyses/blob/master/analyses/notebooks/ESTIMATE%20immune%20score.ipynb?hyper=estimate_immune_score_benefit); no DCB [263.49 (range -1100.78-1734.28)](https://github.com/hammerlab/bladder-analyses/blob/master/analyses/notebooks/ESTIMATE%20immune%20score.ipynb?hyper=estimate_immune_score_no_benefit) ([n=26, Mann-Whitney p=0.33](https://github.com/hammerlab/bladder-analyses/blob/master/analyses/notebooks/ESTIMATE%20immune%20score.ipynb?hyper=estimate_immune_score_mw)).

##

##

## S4D Fig


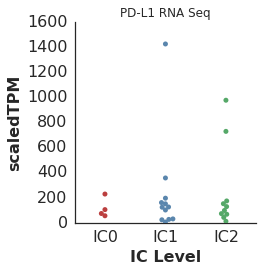


PD-L1 expression as measured by RNA-seq was not associated with PD-L1 IC level ([n=26, Spearman rho=0.045 p=0.83](https://github.com/hammerlab/bladder-analyses/blob/master/analyses/notebooks/Kallisto%2BTximport%20Exhaustion%20Markers.ipynb?hyper=tumor_pdl1_vs_ic_level_spearmanr)). Tumor cell PD-L1 staining was not available.

S4E Fig

##
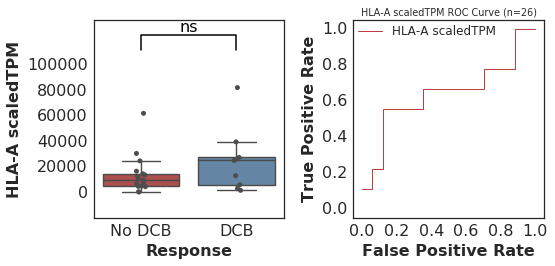

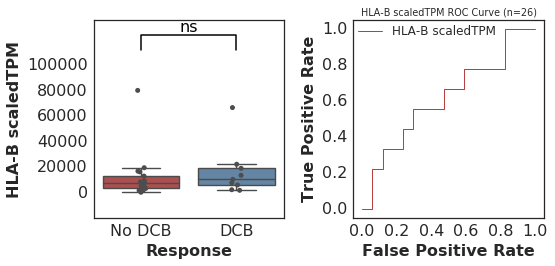

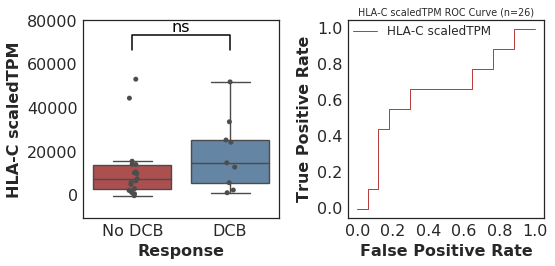


HLA Class I expression was not associated with DCB (HLA-A: [n=26, Mann-Whitney p=0.26](https://github.com/hammerlab/bladder-analyses/blob/master/analyses/notebooks/Kallisto%2BTximport%20HLA%20Expression.ipynb?hyper=HLA-A_mw), HLA-B: [n=26, Mann-Whitney p=0.36](https://github.com/hammerlab/bladder-analyses/blob/master/analyses/notebooks/Kallisto%2BTximport%20HLA%20Expression.ipynb?hyper=HLA-B_mw), HLA-C: [n=26, Mann-Whitney p=0.24](https://github.com/hammerlab/bladder-analyses/blob/master/analyses/notebooks/Kallisto%2BTximport%20HLA%20Expression.ipynb?hyper=HLA-C_mw)).

##

## S4F Fig


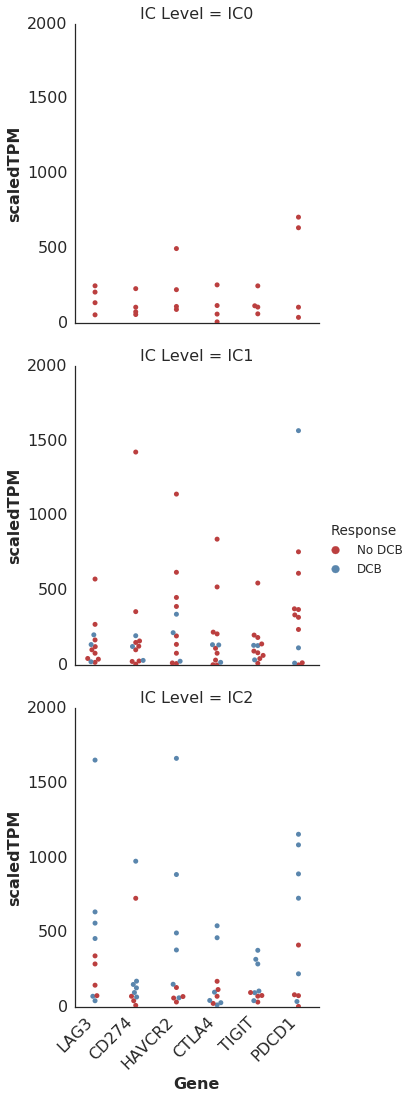


Expression of other inhibitory markers, in particular *HAVCR2* (also known as *TIM-3*) was higher in DCB patients in the IC2 group.

## S4G Fig


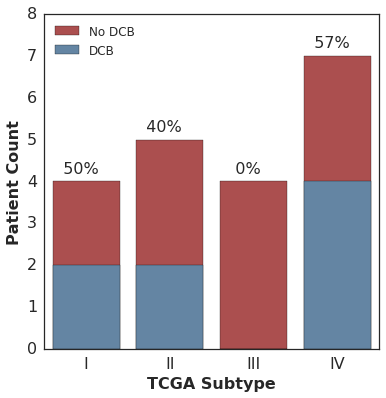


No association was found between TCGA RNA Subtype and response in this sub-study ([n=20, Fisher's Exact p=0.36](https://github.com/hammerlab/bladder-analyses/blob/master/analyses/notebooks/TCGA%20RNA%20Subtypes%20vs.%20Benefit.ipynb?hyper=four_clusters_fisher)).
