# Supplementary material for: Contribution of systemic and somatic factors to clinical response and resistance to PD-L1 blockade in urothelial cancer: An exploratory multi-omic analysis
Source: PLoS Med. 2017 May 26;14(5):e1002309. doi: 10.1371/journal.pmed.1002309 (PMC5446110; doi:10.1371/journal.pmed.1002309)
Supplement: S5 Fig — (A) The hazard associated with log(missense single nucleotide variant [SNV] count per megabase) by level of immune cell (IC) (IC0, IC1, or IC2) programmed death-ligand 1 (PD-L1) expression. (B) The hazard associated with log(missense SNV count per megabase) by presence or absence of liver metastasis at enrollment. (C) The association of peripheral T cell receptor (TCR) clonality prior to treatment with time to mortality (overall survival [OS]) varies according to IC (IC0, IC1, or IC2) PD-L1 expression. (D) The association of peripheral TCR clonality prior to treatment with durable clinical benefit (DCB) varies according to IC (IC0, IC1, or IC2) PD-L1 expression. (E) The association of peripheral TCR clonality prior to treatment with DCB (OS) varies according to IC (IC0, IC1, or IC2) PD-L1 expression. (F) There was no significant relationship between 5-Factor score and pretreatment TCR clonality (n = 26, Spearman rho = 0.25 p = 0.22). (G) Multivariate survival analysis of various clinical, peripheral, and intratumoral biomarkers for association with time to mortality (OS), utilizing a varying-coefficient model, which allows the hazard associated with a 1-unit increase in a biomarker’s value to vary according to the level of intratumoral PD-L1 expression (IC score). Note that the x-axis has been truncated at a value of 10 for clarity, even though this results in the exclusion of some estimated hazard ratio (HR) values (specifically that for pretreatment peripheral TCR clonality among IC2 patients). (DOCX) [file pmed.1002309.s007.docx]

# S5 Fig

## S5A Fig


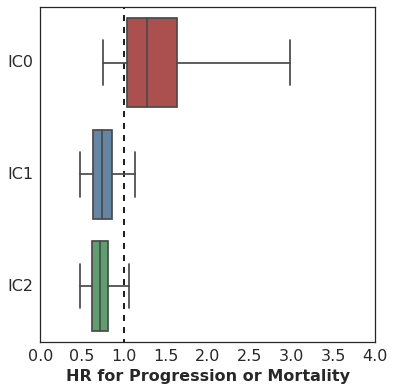


Hazard associated with log(missense SNV count per megabase) by level of immune cell (IC0, IC1 or IC2) PD-L1 expression.

##

## S5B Fig


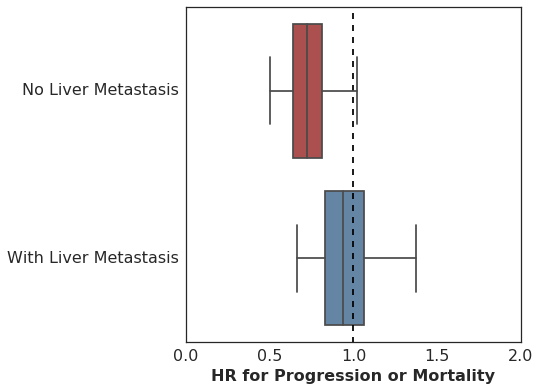


Hazard associated with log(missense SNV count per megabase) by presence or absence of liver metastasis at enrollment.

## S5C Fig


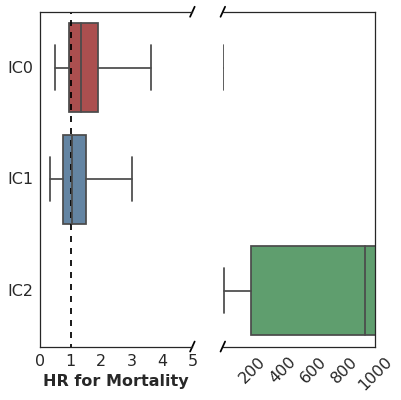


Association of peripheral TCR clonality prior to treatment with time to mortality (OS) varies according to immune cell (IC0, IC1 or IC2) PD-L1 expression.

##

## S5D Fig


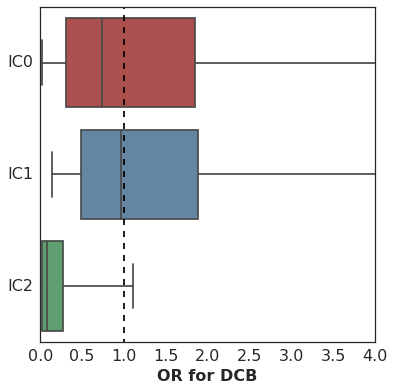


Association of peripheral TCR clonality prior to treatment with DCB varies according to immune cell (IC0, IC1 or IC2) PD-L1 expression.

##

## S5E Fig


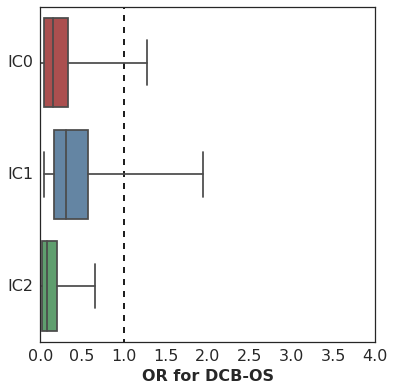


Association of peripheral TCR clonality prior to treatment with DCB (OS) varies according to immune cell (IC0, IC1 or IC2) PD-L1 expression.

## S5F Fig


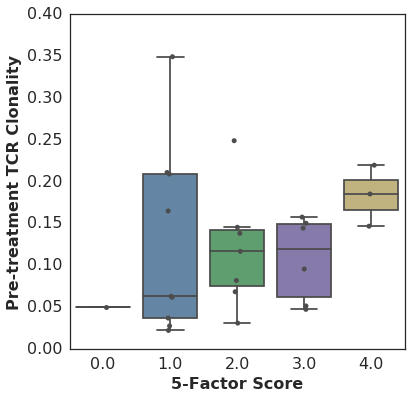


There was no significant relationship between 5-Factor score and pre-treatment TCR clonality ([n=26, Spearman rho=0.25 p=0.22](https://github.com/hammerlab/bladder-analyses/blob/master/analyses/notebooks/Explore%20TCR%20Clonality%20vs.%205-Factor.ipynb?hyper=five_factor_vs_tcr_spearmanr)).

## S5G Fig


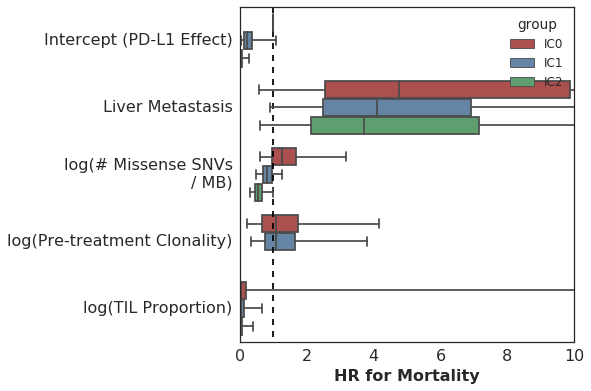


Multivariate survival analysis of various clinical, peripheral and intratumoral biomarkers for association with time to mortality (OS), utilizing a varying-coefficient model which allows the hazard associated with a one-unit increase in a biomarker’s value to vary according to level of intratumoral PD-L1 expression (IC score). Note that the x-axis has been truncated at a value of 10 for clarity even though this results in the exclusion of some estimated HR values (specifically that for pre-treatment peripheral TCR clonality among IC2 patients).
