# Supplementary material for: Contribution of systemic and somatic factors to clinical response and resistance to PD-L1 blockade in urothelial cancer: An exploratory multi-omic analysis
Source: PLoS Med. 2017 May 26;14(5):e1002309. doi: 10.1371/journal.pmed.1002309 (PMC5446110; doi:10.1371/journal.pmed.1002309)
Supplement: S6 Fig — (A) No significant association between the number of missense single nucleotide variants (SNVs) found on MSK-IMPACT and durable clinical benefit (DCB) (DCB 0.13 (range 0.00–0.31) versus no DCB 0.046 (range 0.00–0.37) (n = 25, Mann-Whitney p = 0.42). (B) No significant association between the number of missense SNV found on MSK-IMPACT and overall survival (OS) (survival greater than 12 months 0.093 [range 0.00–0.31] versus less than 12 months 0.074 [range 0.00–0.37]) (n = 25, Mann-Whitney p = 0.78). (C) There was no significant difference in apolipoprotein B mRNA editing enzyme, catalytic polypeptide-like (APOBEC) signature found in tumors from patients with progression-free survival (PFS) DCB (0.19 [range 0.00–0.56]) as compared to no DCB (0.00 [range 0.00–0.46]) (n = 25, Mann-Whitney p = 0.23). (D) There was a significant correlation between missense SNV count and APOBEC signature mutations (n = 25, Pearson r = 0.40 p = 0.048). (E) There was no significant association between FGFR3 mutations or expression (n = 26, Mann-Whitney p = 0.39). (F) There was no significant association between MYC expression and outcome measured by PFS DCB (n = 26, Mann-Whitney p = 0.87). (G) There was no significant association between DDR mutations rated as “possible” by PolyPhen and DCB (n = 25, Mann-Whitney p = 0.20). (H) Univariate association of exonic SNV, missense SNV, and neoepitope load with DCB, with (blue bars) and without (red bars) filtering by expression. (I) Univariate association of exonic SNV, missense SNV, and neoepitope load with OS greater than 12 months, with (blue bars) and without (red bars) filtering by expression. (J) Univariate association of exonic SNV, missense SNV, and neoepitope load with PFS, showing results with (blue bars) and without (red bars) filtering by expression. (K) Univariate association of exonic SNV, missense SNV, and neoepitope load with OS, showing results with (blue bars) and without (red bars) filtering by expression. (L) Univariate associat [file pmed.1002309.s008.docx]

# S6 Fig

## S6A Fig


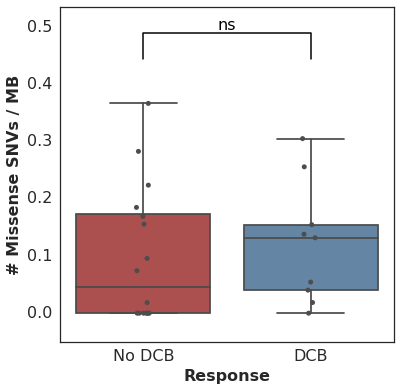


No significant association between the number of missense SNV found on MSK-IMPACT and DCB (DCB [0.13 (range 0.00-0.31)](https://github.com/hammerlab/bladder-analyses/blob/master/analyses/notebooks/Missense%20Filtered%20by%20IMPACT%20Genes.ipynb?hyper=pfs_impact_missense_snv_count_benefit) versus no DCB [0.046 (range 0.00-0.37)](https://github.com/hammerlab/bladder-analyses/blob/master/analyses/notebooks/Missense%20Filtered%20by%20IMPACT%20Genes.ipynb?hyper=pfs_impact_missense_snv_count_no_benefit)

([n=25, Mann-Whitney p=0.42](https://github.com/hammerlab/bladder-analyses/blob/master/analyses/notebooks/Missense%20Filtered%20by%20IMPACT%20Genes.ipynb?hyper=pfs_impact_missense_snv_count_mw)).

## S6B Fig


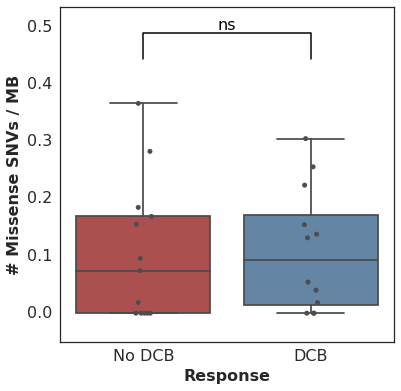


No significant association between the number of missense SNV found on MSK-IMPACT and overall survival (survival greater than 12 months [0.093 (range 0.00-0.31)](https://github.com/hammerlab/bladder-analyses/blob/master/analyses/notebooks/Missense%20Filtered%20by%20IMPACT%20Genes.ipynb?hyper=os_impact_missense_snv_count_benefit), versus less than 12 months [0.074 (range 0.00-0.37)](https://github.com/hammerlab/bladder-analyses/blob/master/analyses/notebooks/Missense%20Filtered%20by%20IMPACT%20Genes.ipynb?hyper=os_impact_missense_snv_count_no_benefit) ([n=25, Mann-Whitney p=0.78](https://github.com/hammerlab/bladder-analyses/blob/master/analyses/notebooks/Missense%20Filtered%20by%20IMPACT%20Genes.ipynb?hyper=os_impact_missense_snv_count_mw)).

## S6C Fig


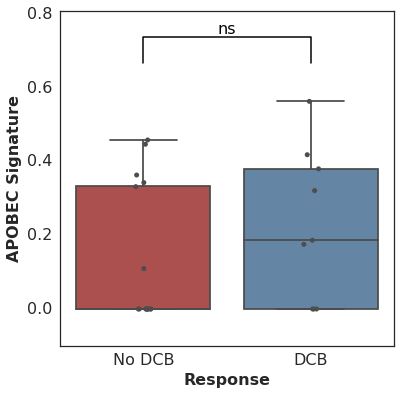


There was no significant difference in APOBEC signature found in tumors from patients with PFS DCB ([0.19 (range 0.00-0.56)](https://github.com/hammerlab/bladder-analyses/blob/master/analyses/notebooks/Signatures%20-%20APOBEC%2C%20ERCC2%2C%20Smoking.ipynb?hyper=apobec_signature_benefit_benefit)) as compared to no DCB ([0.00 (range 0.00-0.46)](https://github.com/hammerlab/bladder-analyses/blob/master/analyses/notebooks/Signatures%20-%20APOBEC%2C%20ERCC2%2C%20Smoking.ipynb?hyper=apobec_signature_benefit_no_benefit)) ([n=25, Mann-Whitney p=0.23](https://github.com/hammerlab/bladder-analyses/blob/master/analyses/notebooks/Signatures%20-%20APOBEC%2C%20ERCC2%2C%20Smoking.ipynb?hyper=apobec_signature_benefit_mw)).

##

##

## S6D Fig


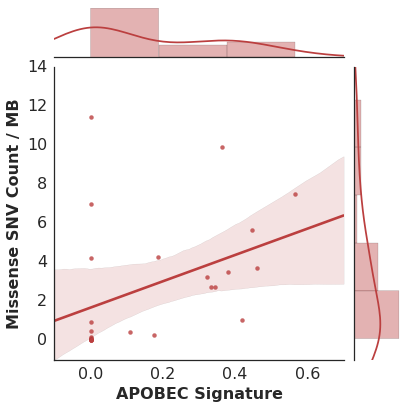


There was a significant correlation between missense SNV count and APOBEC signature mutations ([n=25, Pearson r=0.40 p=0.048](https://github.com/hammerlab/bladder-analyses/blob/master/analyses/notebooks/Signatures%20-%20APOBEC%2C%20ERCC2%2C%20Smoking.ipynb?hyper=apobec_vs_missense_pearsonr)).

##

##

## S6E Fig
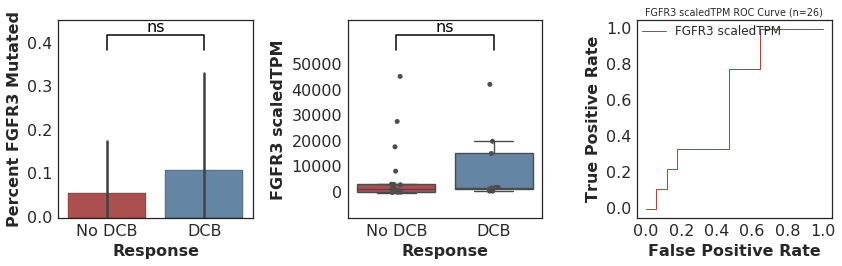


There was no significant association between FGFR3 mutations or expression ([n=26, Mann-Whitney p=0.39](https://github.com/hammerlab/bladder-analyses/blob/master/analyses/notebooks/Kallisto%2BTximport%20JAK1%2CJAK2%2CB2M%2CERCC2%2CFGFR3%2CWNT7B.ipynb?hyper=FGFR3_mw)).

## S6F Fig


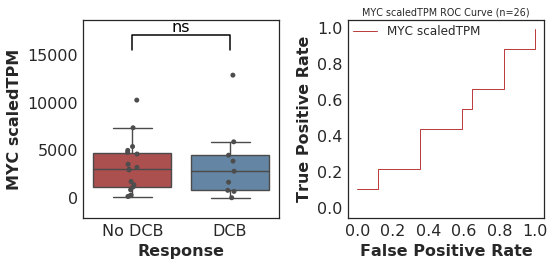


There was no significant association between MYC expression and outcome measured by PFS DCB ([n=26, Mann-Whitney p=0.87](https://github.com/hammerlab/bladder-analyses/blob/master/analyses/notebooks/Kallisto%2BTximport%20JAK1%2CJAK2%2CB2M%2CERCC2%2CFGFR3%2CWNT7B.ipynb?hyper=MYC_mw)).

##

##

## S6G Fig


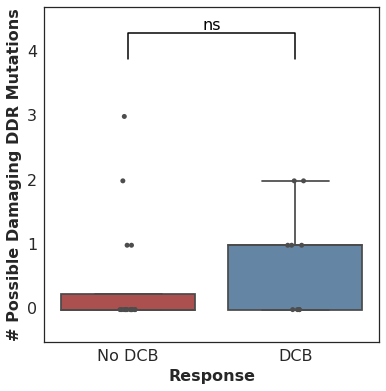


There was no significant association between DNA damage response (DDR) mutations rated as “possible” by PolyPhen and DCB ([n=25, Mann-Whitney p=0.20](https://github.com/hammerlab/bladder-analyses/blob/master/analyses/notebooks/DNA%20Damage%20Repair%20with%20Matt%27s%20DDR%20List.ipynb?hyper=num_ddr_possibly_mw)).

## S6H Fig


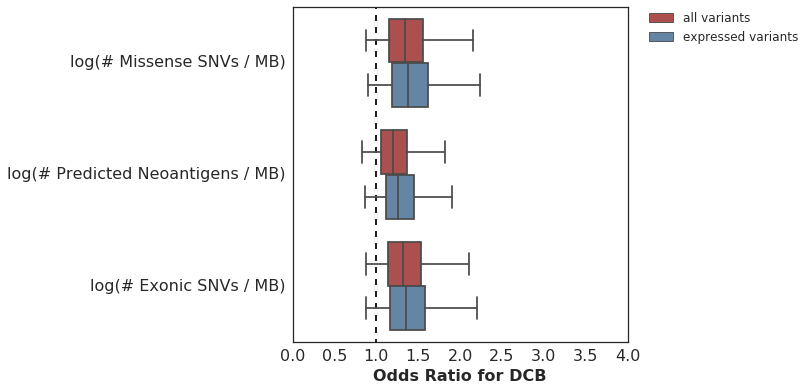


Univariate association of exonic SNV, missense SNV and neoepitope load with DCB, with (blue bars) and without (red bars) filtering by expression.

##

##

## S6I Fig


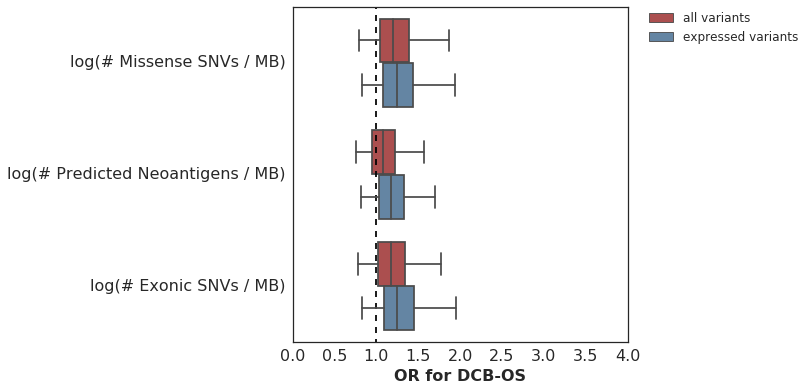


Univariate association of exonic SNV, missense SNV and neoepitope load with OS greater than 12 months, with (blue bars) and without (red bars) filtering by expression.

##

##

## S6J Fig


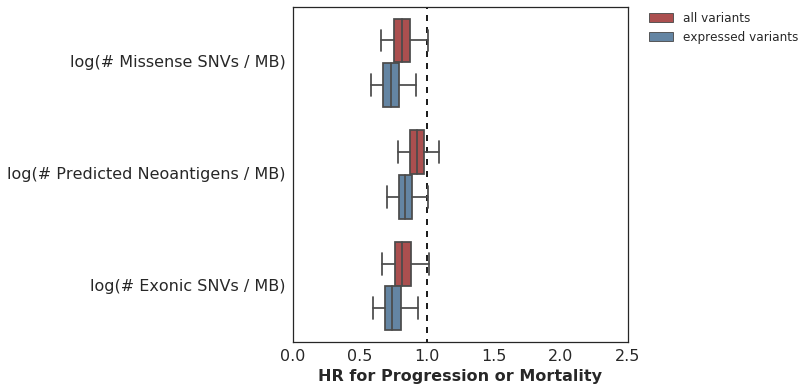


Univariate association of exonic SNV, missense SNV and neoepitope load with PFS, showing results with (blue bars) and without (red bars) filtering by expression.

##

##

## S6K Fig


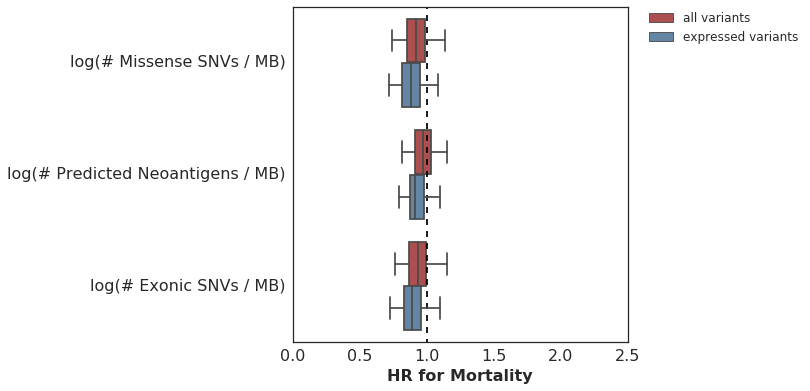


Univariate association of exonic SNV, missense SNV and neoepitope load with OS, showing results with (blue bars) and without (red bars) filtering by expression.

## S6L Fig


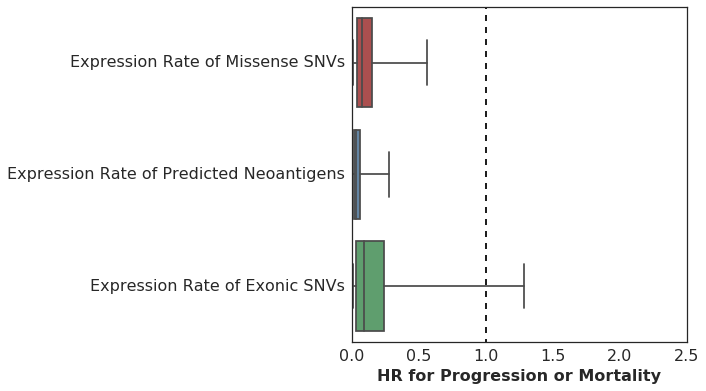


Univariate association of expressed/total ratio for exonic SNV, missense SNV, and neoantigen loads with PFS.

##

##

## S6M Fig


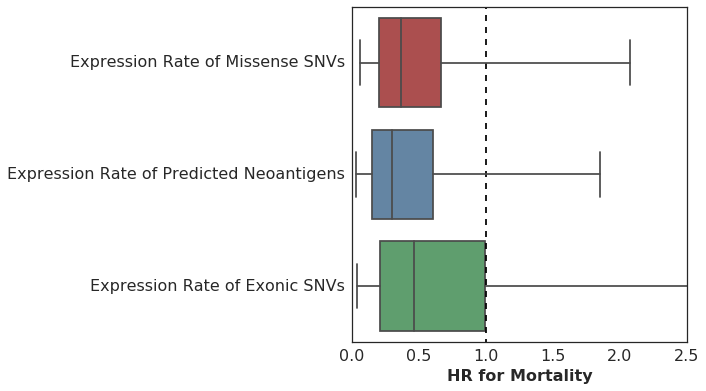


Univariate association of expressed/total ratio for exonic SNV, missense SNV, and neoantigen loads with OS.

## S6N Fig


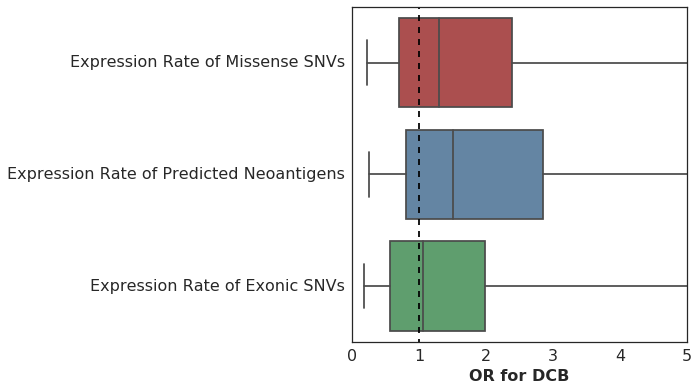


Univariate association of expressed/total ratio for exonic SNV, missense SNV, and neoantigen loads with DCB (PFS greater than 12 months).

##

##

## S6O Fig


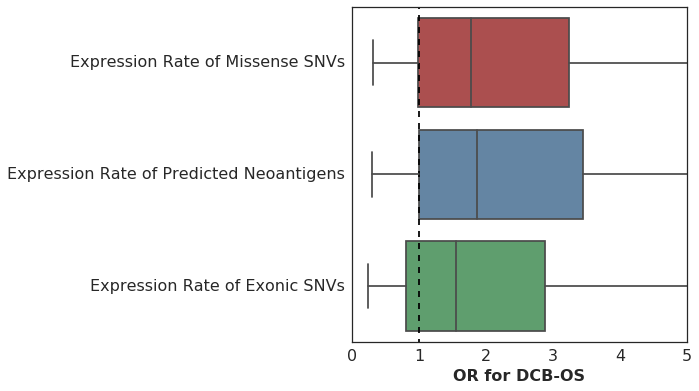


Univariate association of expressed/total ratio for exonic SNV, missense SNV, and neoantigen loads with OS greater than 12 months.

##

## S6P Fig


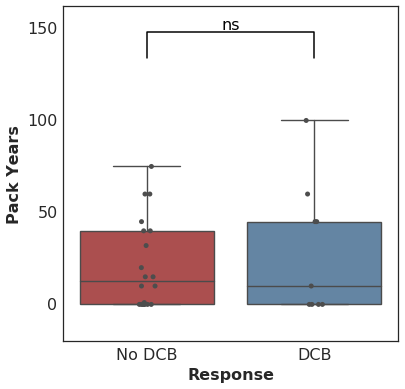


There was no significant association between pack years of reported smoking history and DCB ([n=29, Mann-Whitney p=0.87](https://github.com/hammerlab/bladder-analyses/blob/master/analyses/notebooks/Supp%20Figure%20Extra%20Clinical%20Factors%20Pkyrs%2C%205-factor.ipynb?hyper=packyears_mw)).

##

## S6Q Fig


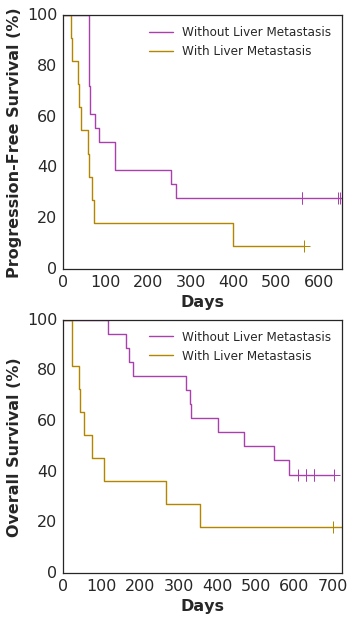


There were significant associations between PFS ([n=29, log-rank p=0.024](https://github.com/hammerlab/bladder-analyses/blob/master/analyses/notebooks/Supp%20Figure%20Extra%20Clinical%20Factors%20Pkyrs%2C%205-factor.ipynb?hyper=liver_pfs_logrank)) and OS ([n=29, log-rank p=0.018](https://github.com/hammerlab/bladder-analyses/blob/master/analyses/notebooks/Supp%20Figure%20Extra%20Clinical%20Factors%20Pkyrs%2C%205-factor.ipynb?hyper=liver_os_logrank)) and the presence of liver metastasis.

## S6R Fig


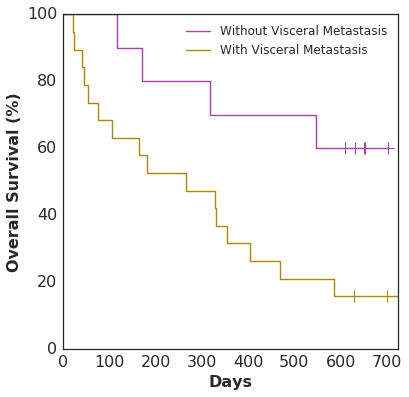


There was a significant association between the presence of visceral metastases and poor overall survival ([n=29, log-rank p=0.020](https://github.com/hammerlab/bladder-analyses/blob/master/analyses/notebooks/Supp%20Figure%20Extra%20Clinical%20Factors%20Pkyrs%2C%205-factor.ipynb?hyper=visceral_os_logrank)).

## S6S Fig


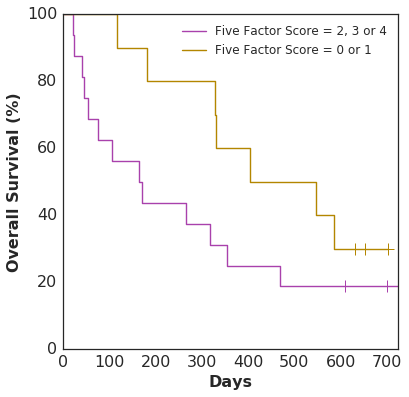


There was not a significant association between 5-factor score and OS ([n=26, log-rank p=0.13](https://github.com/hammerlab/bladder-analyses/blob/master/analyses/notebooks/Supp%20Figure%20Extra%20Clinical%20Factors%20Pkyrs%2C%205-factor.ipynb?hyper=fivefactor_os_logrank)).

## S6T Fig


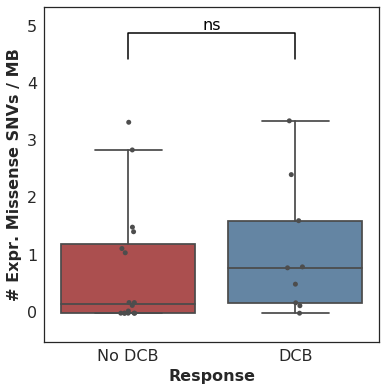


No significant association between the number of expressed missense SNV per megabase and DCB (DCB [0.79 (range 0.00-3.36)](https://github.com/hammerlab/bladder-analyses/blob/master/analyses/notebooks/Count%20Plots.ipynb?hyper=pfs_expressed_missense_snv_count_benefit), versus no DCB: [0.16 (range 0.00-3.34)](https://github.com/hammerlab/bladder-analyses/blob/master/analyses/notebooks/Count%20Plots.ipynb?hyper=pfs_expressed_missense_snv_count_no_benefit)), [n=25, Mann-Whitney p=0.26](https://github.com/hammerlab/bladder-analyses/blob/master/analyses/notebooks/Count%20Plots.ipynb?hyper=pfs_expressed_missense_snv_count_mw).
