# Supplementary material for: Contribution of systemic and somatic factors to clinical response and resistance to PD-L1 blockade in urothelial cancer: An exploratory multi-omic analysis
Source: PLoS Med. 2017 May 26;14(5):e1002309. doi: 10.1371/journal.pmed.1002309 (PMC5446110; doi:10.1371/journal.pmed.1002309)
Supplement: S1 Text — (DOCX) [file pmed.1002309.s009.docx]

## **S1 STROBE Criteria**

In the interest of being thorough, we have included here each of the items on the STROBE checklist with our comments. This is part of a response to issue 0.5 raised by the editors.

### Title & Abstract

1a. Indicate study's design with a commonly used term in the title or abstract

> The abstract and title have been updated to describe this analysis as an Exploratory analysis.

1b. provide in the abstract an informative and balanced summary of what was done and what was found

> We have included mention of the limited sample size in the abstract, as requested

####

### Introduction

2. Explain the background and rationale for the investigation being reported

> This is included in the Introduction of the manuscript

3. State specific objectives, including any pre-specified hypotheses

> We have included our pre-specified analysis plan as a supplementary file, and have included the statement of our study aims / hypothesis in the Introduction.

### Methods

4. Present key elements of study design early in the paper

> These are provided in the first heading of the methods section.

5. Describe the setting, locations and relevant dates including periods of recruitment, exposure, follow-up and data collection

> These were provided in the manuscript describing the larger cohort, IMVigor 210.

6. Cohort study: give the eligibility criteria and the sources and methods of selection of participants. Describe methods of follow-up.

> The Methods states that “All patients had locally advanced or metastatic urothelial carcinoma and were treated at Memorial Sloan Kettering Cancer Center (n=[29](https://github.com/hammerlab/bladder-analyses/blob/master/analyses/notebooks/Patient%20Numbers.ipynb?hyper=number_all_patients)) on protocol NCT02108652.”

7. Clearly define all outcomes, exposures, predictors, potential confounders, and effect modifiers. Give diagnostic criteria, if applicable.

> The measurement of each outcome measure, predictor, potential confounder or effect modifier was clearly defined. Of these, variables treated as outcomes are separated from those which are not; the pre-specified analysis plan further distinguishes between variables which are supposed effect modifiers, supposed confounders and possible predictors. The study design does not allow distinction between these three classes of covariates.

8. For each variable of interest, give sources of data and details of methods of assessment (measurement). Describe comparability of assessment methods if there is more than one group.

> The data sources and methods of measurement are detailed in the Methods section for each variable of interest.

9. Describe any efforts to address potential sources of bias.

> We describe in the paper how we performed a multivariate analysis to adjust for prognostic indicators in our analysis.

10. Explain how the study size was arrived at.

> This is detailed in the Methods section, in conjunction with the study design. The analysis is limited to patients enrolled in IMvigor210 at a single institution, who gave informed consent for correlative analysis and whose samples were of adequate quality to permit analysis.

11. Explain how quantitative variables were handled in the analyses. If applicable, describe which groupings were chosen, and why.

> In some cases, quantitative variables were grouped into sets above/below the median for illustrative purposes. For our outcome variable the grouping was selected in our pre-specified analysis plan.

12. Statistical methods:

- describe all statistical methods, including those used to control for confounding

- describe any methods used to examine subgroups and interactions

- explain how missing data were addressed

- cohort study: if applicable, explain how loss to follow-up was addressed

- describe any sensitivity analyses

> The statistical methods section of the manuscript details many of these issues. The issue of “loss to follow-up” is addressed through the use of survival analysis, and subjects with missing data are excluded from relevant analyses.

### Results

13. Participants:

- report numbers of individuals at each stage of study. e.g. numbers potentially eligible, examined for eligibility, confirmed eligible, included in the study, completing follow-up, and analyzed.

- give reasons for non-participation at each stage.

- consider use of a flow diagram

> The sample size for each quantitative result cited in the paper is provided, as well as the numbers of patients eligible, with DNA, with RNAseq, and with TCR-seq data available. Those samples that were excluded for any reason are now specifically named in the Methods.

14. Descriptive:

- Give characteristics of study participants and information on exposures and potential confounders.

- Indicate number of participants with missing data for each variable of interest

- Summarize follow-up time (e.g. average and total amount)

> These are included in Table 1.

15. Outcome: Report numbers of outcome events or summary measures over time

> These are included in Table 1.

16. Main results:

- Give unadjusted estimates and, if applicable, confounder-adjusted estimates and their precision (e.g. 95% CI). Make clear which confounders were adjusted for and why they were included.

- Report category boundaries when continuous variables were categorized

- If relevant, consider translating estimates of relative risk into absolute risk for a meaningful time period

> We have reported these details with our results.

17. Other analyses: Report other analyses done -- e.g. analyses of subgroups and interactions, and sensitivity analyses.

> Sensitivity analyses are discussed in greater detail in our GitHub repository.

### Discussion

18. Key results: summarize key results with reference to study objectives.

> This summary is provided in the first section of our discussion.

19. Discuss limitations of the study, taking into account sources of potential bias or imprecision. Discuss both direction & magnitude of any potential bias.

> We have edited this section to include stronger language regarding implications of sample size, in line with recommendation from Reviewer #1. We wish to thank this reviewer for specifically mentioning potential for Type 1 error.

20. Give a cautious overall interpretation of results considering objectives, limitations, multiplicity of analyses, results from similar studies, and other relevant evidence.

> In light of reviewer comments, we have increased the level of caution in our findings and more strongly recommended replication studies.

21. Generalizability: Discuss the generalizability (external validity) of the study results.

> This is a hypothesis-generating analysis, and so no generalizability should be assumed.

### Other information

22. Funding: give the source of funding & the role of the funders for the present study and, if applicable, for the original study on which the present article is based.

> This information is provided at the end of the manuscript.
