# Supplementary material for: Contribution of systemic and somatic factors to clinical response and resistance to PD-L1 blockade in urothelial cancer: An exploratory multi-omic analysis
Source: PLoS Med. 2017 May 26;14(5):e1002309. doi: 10.1371/journal.pmed.1002309 (PMC5446110; doi:10.1371/journal.pmed.1002309)
Supplement: S2 Text — (DOCX) [file pmed.1002309.s010.docx]

# S1 Pre-Specified Analysis Plan

The following text is drawn from the grant which primarily funded this study; text is presented here with some details redacted. For the most part, redacted components are those which pertain to other analyses, some of which are ongoing. Removed portions are indicated by [...].

**Specific Aims:**

**Aim 1. Determine the mutational load and signatures associated with response and clinical benefit to checkpoint blockade in urothelial cancer.** DNA from tumors and matched normal blood will undergo whole exome sequencing (WES), followed by analysis for mutational burden^6^ and the APOBEC signature.^9^ We hypothesize that mutational burden and/or APOBEC signature are associated with benefit from anti-PD-L1 therapy [... details about additional studies redacted].

**Aim 2. Define the predicted neoantigens and T cell receptor (TCR) diversity in urothelial cancer.**  Neoantigens, tumor peptides resulting from somatic mutations and recognized by the immune system, will be predicted *in silico* based on WES data and a subset validated *in vitro*. TCR sequencing will be conducted on tumors and peripheral blood collected at baseline and at three post-treatment time points.

**Methods:**

**Aim 1: Determine the mutational load and signatures associated with response and clinical benefit to checkpoint blockade in urothelial cancer.**

**1A. Determinants of clinical benefit.** Baseline evaluations for all patients include either abdominal and pelvic computed tomography (CT) scans or magnetic resonance imaging (MRI) for abdominal or pelvic indicator lesions. Up to five indicator lesions are assessed by a reference radiologist prior to treatment and again at 6-9 week intervals dependent on individual trial requirements. Response will be assessed using Response Evaluation Criteria in Solid Tumors (RECIST) version 1.1 and classified as complete response (CR), partial response (PR), stable disease (SD), or progression of disease (POD). PR is defined as at least a 30% decrease in indicator lesion measurements from baseline and progression is defined as at least a 20% increase in indicator lesions from the nadir sum of measurements. Clinical Benefit will be defined as progression-free at 6 months, twice the PFS seen with conventional treatments. Progression-free survival (PFS) will be defined as the time from first dose of immunotherapy to the first documented objective tumor progression, or last scan date for patients who did not progress, and will be measured as both a time to event variable and as a dichotomous variable of progression-free status at 6 months (PFS-6), previously used for correlations in a prior melanoma study.^6^

**1B. Tumor acquisition.** Tumors have already been collected from 29 patients on a trial of MPDL3280A in metastatic urothelial cancer (HBUC No. HBS2014082, IRB Waiver WA0523-14) . Acquisition is planned for upcoming trials of pembrolizumab and MPDL3280A in both previously treated and chemotherapy-naïve patient populations (Table 1).

**1C. Determine if mutational burden correlates with clinical benefit from checkpoint blockade therapy.** After DNA isolation (Qiagen), samples will undergo whole exome sequencing (MSKCC Genomics Core) using the SureSelect Human All Exon 50MB kit (Agilent) for exon capture and sequencing on the HiSeq 2000 platform (Illumina) to a goal of 100X coverage for frozen samples and 150X for FFPE samples. Raw sequencing data will be mapped to the human reference genome. Alignment, base-quality score recalibration, duplicate-read removal, and exclusion of germline variants will be performed using the Genome Analysis Toolkit (GATK).^10^ Known single nucleotide polymorphisms^11-13^ will be eliminated, except those with a normal allelic fraction of zero. Mutations will be annotated using SnpEffect,^14^ called with commonly-used callers including Somatic Sniper,^15^ VarScan,^16^ Strelka^17^ and MuTect^18^, then filtered and manually reviewed using IGV.^19^ This analysis will generate a list of somatic nonsynonymous exonic mutations and indels found in each tumor. Total exonic nonsynonymous missense mutational burden, as well as total somatic variant burden (including insertions and deletions) will be compared for tumors in patients with and without benefit from checkpoint blockade therapies. In light of the suggestive findings of POLE and other mutations in DNA repair and hypermutator genes, we will also ascertain whether such mutations are likely to be deleterious^20^ and thus potentially leading to an elevated mutational burden.

**1D. Determine if the APOBEC signature correlates with clinical benefit from checkpoint blockade therapy.** Whereas the majority of cutaneous melanomas harbor an ultraviolet damage signature at the DNA level^8^ and a subset of lung cancers exhibit a smoking signature,^21^ the APOBEC signature characterizes approximately one third of urothelial cancers.^9,22^ The APOBEC (apolipoprotein B mRNA-editing enzyme, catalytic polypeptide-like) proteins are part of the cytidine deaminase family that converts cytosine to uracil. APOBECs are thought to play a role in innate immunity; one of their functions is to hypermutate retrovirus’s proviral DNA, thereby impairing their function. In tumors, however, they cause a characteristic mutational pattern: a change to T or G at TCW motifs (where W is T or A), resulting in TTW or TGW motifs. Using an algorithm established at the NIH,^22^ we will analyze our samples for the APOBEC signature and assess whether its presence is associated with clinical benefit from checkpoint blockade therapies.

**Aim 2. Define the predicted neoantigens and T cell receptor (TCR) diversity in urothelial cancer.**

**2A. Measure predicted neoantigens and compare their number in tumors with and without response to checkpoint blockade therapy.** We hypothesize that an increase in predicted neoantigens will correlate with clinical benefit from checkpoint blockade therapy. Using the sequence data acquired in Aim 1B, candidate neoantigens will be identified using methods developed by our group.^6^ First, ATHLATES (http://www.broadinstitute.org/scientific-community/science/projects/viral-genomics/athlates) is used to determine the patients’ MHC Class I HLA types.^23^ NASeek, a software program developed by our group, is then used to “virtually translate” a 17-amino acid window surrounding the amino acid altered by each mutation. Next, we will use NetMHC^24^ (http://www.cbs.dtu.dk/services/NetMHC/), a program used widely in the field,^25,26^ to predict which 9-amino acid window binds best to the patient’s own MHC Class I. We will then compare the predicted neoantigen load between tumors from patients with and without clinical benefit. To improve upon the predictions of NetMHC, we will also search for “TCR recognition motifs” as described in studies by Garcia and Davis.^27,28^ We have improved our bioinformatic algorithm from our recently published work^6^ in order to analyze the similarity of each predicted neoantigen to antigens with a documented T cell or interferon gamma response measured *in vitro* and catalogued in the Immune Epitope Database (IEDB).^29^ The current algorithm incorporates a Blosum matrix for amino acid similarity, and allows for discontinuous stretches of similarity in order to compare the entire presented nonamer and putative TCR contact residues rather than simply a continuous substring (as in our prior algorithm).^6^ The updated program permits but does not require comparison between tumors; rather, it shifts the programmatic emphasis to patterns of TCR contact residues that are more likely, based on published *in vitro* data, to elicit a cytotoxic T cell response.

**2B. Validate predicted neoantigens *in vitro*.** The validation of predicted neoantigens remains a significant challenge in this field. In one study, a prominent lab was able to detect T cells specific for only a single neoantigen out of 450 potential neoantigens (predicted from identified and expressed mutations) screened from a patient treated with ipilimumab.^30^ The presumed activity of MPDL3280A at the tumor site may make detection of neoantigen-specific T cells in the peripheral blood even more challenging. Accordingly, we will continue to work on improving the sensitivity of our assays and to employ novel approaches (i.e. we are presently working to incorporate novel technologies in our laboratory, such as the tetramer technology developed by Ton Schumacher,<http://www.immudex.com/>). We propose to screen 6 MPDL3280A patients (3 complete responders and 3 non-responders) for neoantigen-specific T cell responses in banked PBMC samples. As an initial approach, we will use synthetic peptides to stimulate banked patient lymphocytes followed by intracellular cytokine staining, as previously described.^6^ We will stimulate autologous peripheral blood mononuclear cells (PBMC) with synthetic peptides corresponding to predicted neoantigens, followed by flow cytometry and intracellular cytokine staining for CD8, CD4, interferon gamma, TNF-alpha, MIP-1 beta and CD107a. As an alternative and complementary approach, we will use tetramer technology, in which a synthetic peptide neoantigen is pre-loaded onto a specific MHC tetramer. If we are successful at detecting tetramer positive cells, we will characterize the phenotype of these cells by flow cytometry and functional assays, as previously described by our group. ^31,32^

**2C. Measure changes in TCR clonality.** A major unanswered question in the field of immunotherapy is whether an effective anti-tumor response requires T cell activation against a multitude of tumor neoantigens, *versus* a subset of T cells reacting powerfully against a small number of neoantigens. While TCR sequencing cannot identify the peptide(s) that a given T cell recognizes, it can serve as a proxy for T cell diversity. We have gathered PBMC prior to treatment and at 3 time points after treatment from 29 patients treated with MPDL3280A [... details about additional studies redacted]. In addition, we have allocated tumor sections for intratumoral TCR sequencing. Through collaboration with Adaptive Biotechnologies (<http://www.adaptivebiotech.com/>), we will sequence the variable region of the TCR beta chain of T cells in the banked samples from the MPDL3280A-treated patients (with a plan to extend this collaboration to apply to other trials). Clonality is portrayed as Shannon entropy, normalized for sample size. It ranges from zero to one, with zero indicating that every T-cell is a unique clone, one indicating that all T-cells are the same clone, and most samples falling within a range of 0.1 to 0.4 (Fig. 3 illustrates a theoretical example of data with TCR measurements from one time point). We will therefore be able to compare pretreatment intratumoral to peripheral TCR diversity, as well as pre-treatment to post-treatment peripheral TCR diversity. The correlation of these data to clinical outcome will elucidate whether a T cell repertoire that is capable of tumor control is (a) diverse and polyclonal or (b) consists of a small number of dominant clones; both alternatives have been reported in small studies, neither in association with exome sequencing data.^33,34^ When integrated with WES results, we hypothesize that an elevated mutational burden *in combination with* T cell clonal dominance in the intratumoral lymphocytes prior to treatment and/or the peripheral T cells after treatment will be necessary for effective tumor control upon treatment with immunotherapy.

**Statistical Considerations**

We conservatively anticipate that about 25% of patients will derive clinical benefit (progression-free at 6 months), assuming PFS is exponentially distributed and median PFS is 3 months. We will correlate clinical benefit with mutational load (count) and APOBEC signature (binary variable) in Aim 1 using the Wilcoxon rank sum and Fisher exact tests, respectively, and with predicted amount of neoantigens (continuous) and TCR diversity (continuous value between 0 and 1) in Aim 2 using the Wilcoxon rank sum test. These methods are non-parametric and are suitable for small samples, thus the analysis will be done within each trial both separately and using a pooled sample of patients from all trials. Assuming an APOBEC signature prevalence of 33%, with 30 patients we will be able to detect 55% difference in clinical benefit rate between patients with the APOBEC signature present or absent with 80% power and 5% 2-sided type 1 error. Similarly, we will be able to detect a 3-fold difference in the mutational load (150 vs. 450 mean number of mutations with standard deviation of 200) in patients with and without clinical benefit with 90% power and 5% 2-sided type 1 error. Predicted amount of neoantigens and TCR diversity will be transformed to follow standard normal distribution (logit transformation will be used for TCR diversity). With 30 patients we will be able to detect the difference of 1.5 in standardized means between patients with and without clinical benefit with 90% power and 5% type 1 error. Power will be even greater for pooled analysis. Trials with 10 informative patients will have smaller power in individual analyses, but still they will increase the power of the pooled analysis. We will also evaluate whether there are any interactions between effects of these biomarkers and treatment regimens, although the power of such tests might be limited. If necessary, we will evaluate the effect of these biomarkers on clinical benefit after adjusting for MSKCC risk score or other clinical confounders using logistic regression. All 4 biomarkers will also be correlated with time to event PFS using Cox proportional hazards model. Since there are only four biomarkers of interest and their effects might be correlated, no adjustment for multiple comparisons will be done.

**Potential Pitfalls and Alternatives:** Although 29 samples are already prepared for sequencing, it is possible that accrual during the grant period will limit the sample size and will not permit validation of our findings. Bladder cancer specialists [... details redacted for confidentiality] are well known to our group and plan to collaborate on this work [... details redacted for confidentiality].

# Deviations from the Pre-Specified Analysis Plan

**1A. Determinants of clinical benefit.**

Upon extensive discussion with practicing clinicians and others, it was decided to look at a second definition of durable clinical benefit defined as overall survival greater than 12 months, in addition to the pre-specified definition of progression-free-survival greater than 6 months. This decision was made out of consideration that improvement in overall survival is the clinical goal of treatment.

In addition, for some analyses and given our relatively small sample size, we furthermore analyzed the data continuously for both endpoints using a survival analysis where time to event (either survival or progression-free-survival) is the outcome. This decision was motivated by two goals: (1) to benefit from the increased power of these methods, in order to lessen type II error and (2) to better understand the time-dependent dynamics of the response to treatment.

Rather than pick and choose from among these results, we felt it was important to highlight results from all 4 variations for each analysis performed. If not in the main document, then results from alternate analyses are included in the Supplement.

Finally, due to known limitations of RECIST and mRECIST criteria, these were not used as clinical outcomes as highlighted in the primary analysis plan.

**1C. Determine if mutational burden correlates with clinical benefit from checkpoint blockade therapy.**

The details of the somatic calling methods were changed from their pre-specified versions. Rather than using SnpEffect,^14^ which combines results from Somatic Sniper,^15^ VarScan,^16^ Strelka^17^ and MuTect^18^, we instead used sets of tools such as Varcode which have been developed more recently. In addition, the somatic mutations were called using only Strelka and MuTect.

**2A. Measure predicted neoantigens and compare their number in tumors with and without response to checkpoint blockade therapy.**

Instead of using ATHLATES to determine HLA type, we used OptiType. In our experience, this tool yields better inference for type I alleles. Also, in a recent study by [Kiyotani et al](http://www.nature.com/jhg/journal/vaop/ncurrent/full/jhg2016141a.html) in the Journal of Human Genetics (https://www.ncbi.nlm.nih.gov/pubmed/26818738), Optitype showed the highest accuracy of 97.2% for HLA class I alleles. Similarly, instead of using NASeek, we used a different pipeline.

**Multivariate analysis**

The selection of which markers to include in the multivariate analysis was modified from the pre-specified analysis plan, given the results of univariate association analyses. The APOBEC signature was not included, for example, since there was little to no observable correlation between this signature and benefit from therapy.

# Post-Hoc Analyses

Several analyses, such as the time-varying effect analysis and the analysis of interaction with PD-L1 expression levels, were not included in the pre-specified analysis plan above. Instead, they were proposed after reviewing the results of those analyses described above but were specified prior to execution in dedicated issues tracked in our internal GitHub repository.

**Time-varying effect analysis**

The initial hypothesis for the time-varying effect analysis was to separate the effect of mutation burden on progression-free survival “after excluding rapid progressors” (patients who progressed in the first 3 months). The analysis was then fleshed out to include the interaction effect with time using this 3 month threshold, as well as the non-parametric analysis to see if the 3 month threshold was a discrete change point.

**Interaction of mutation burden with PD-L1 expression**

Similarly, we hypothesized that the association of biomarkers with overall and progression-free survival in this cohort might vary by PD-L1 expression levels, given that PD-L1 expression had been shown to correlate with clinical benefit from atezolizumab.

**References for Pre-specified Analysis**

1. Sharma P, Shen Y, Wen S, et al. CD8 tumor-infiltrating lymphocytes are predictive of survival in muscle-invasive urothelial carcinoma. Proc Natl Acad Sci U S A 2007;104:3967-72.

2. Powles T, Eder JP, Fine GD, et al. MPDL3280A (anti-PD-L1) treatment leads to clinical activity in metastatic bladder cancer. Nature 2014;515:558-62.

3. Plimack ER, Gupta S, Bellmunt J et al. A phase 1b study of pembrolizumab (Pembro; MK-3475) in patients (Pts) with advanced urothelial tract cancer. Ann Oncol 2014;25:41.

4. Gallagher DJ, Milowsky MI, Gerst SR, et al. Phase II study of sunitinib in patients with metastatic urothelial cancer. J Clin Oncol 2010;28:1373-9.

5. Milowsky MI, Iyer G, Regazzi AM, et al. Phase II study of everolimus in metastatic urothelial cancer. BJU Int 2013;112:462-70.

6. Snyder A, Makarov V, Merghoub T, et al. Genetic basis for clinical response to CTLA-4 blockade in melanoma. N Engl J Med 2014;371:2189-99.

7. Rizvi N, Hellmann M, Snyder A, et al. Mutational Landscape Determines Sensitivity to Programmed Cell Death-1 Blockade in Non-Small Cell Lung Cancer. Manuscript under revision 2015.

8. Alexandrov LB, Nik-Zainal S, Wedge DC, et al. Signatures of mutational processes in human cancer. Nature 2013;500:415-21.

9. Nordentoft I, Lamy P, Birkenkamp-Demtroder K, et al. Mutational context and diverse clonal development in early and late bladder cancer. Cell Rep 2014;7:1649-63.

10. DePristo MA, Banks E, Poplin R, et al. A framework for variation discovery and genotyping using next-generation DNA sequencing data. Nat Genet 2011;43:491-8.

11. Sherry ST, Ward M, Sirotkin K. dbSNP-database for single nucleotide polymorphisms and other classes of minor genetic variation. Genome Res 1999;9:677-9.

12. Server EV. NHLBI GO Exome Sequencing Project (ESP).<http://evs.qs.washington.edu/EVS>

13. Via M, Gignoux C, Burchard EG. The 1000 Genomes Project: new opportunities for research and social challenges. Genome Med 2010;2:3.

14. De Baets G, Van Durme J, Reumers J, et al. SNPeffect 4.0: on-line prediction of molecular and structural effects of protein-coding variants. Nucleic Acids Res 2012;40:D935-9.

15. Larson DE, Harris CC, Chen K, et al. SomaticSniper: identification of somatic point mutations in whole genome sequencing data. Bioinformatics 2012;28:311-7.

16. Koboldt DC, Zhang Q, Larson DE, et al. VarScan 2: somatic mutation and copy number alteration discovery in cancer by exome sequencing. Genome Res 2012;22:568-76.

17. Saunders CT, Wong WS, Swamy S, Becq J, Murray LJ, Cheetham RK. Strelka: accurate somatic small-variant calling from sequenced tumor-normal sample pairs. Bioinformatics 2012;28:1811-7.

18. Cibulskis K, Lawrence MS, Carter SL, et al. Sensitive detection of somatic point mutations in impure and heterogeneous cancer samples. Nat Biotechnol 2013;31:213-9.

19. Robinson JT, Thorvaldsdottir H, Winckler W, et al. Integrative genomics viewer. Nat Biotechnol 2011;29:24-6.

20. Reva B, Antipin Y, Sander C. Predicting the functional impact of protein mutations: application to cancer genomics. Nucleic Acids Res 2011;39:e118.

21. Cancer Genome Atlas Research N. Comprehensive molecular profiling of lung adenocarcinoma. Nature 2014;511:543-50.

22. Roberts SA, Lawrence MS, Klimczak LJ, et al. An APOBEC cytidine deaminase mutagenesis pattern is widespread in human cancers. Nat Genet 2013;45:970-6.

23. Liu C, Yang X, Duffy B, et al. ATHLATES: accurate typing of human leukocyte antigen through exome sequencing. Nucleic Acids Res 2013;41:e142.

24. Lundegaard C, Lamberth K, Harndahl M, et al. NetMHC-3.0: accurate web accessible predictions of human, mouse and monkey MHC class I affinities for peptides of length 8-11. Nucleic Acids Res 2008;36:W509-12.

25. Matsushita H, Vesely MD, Koboldt DC, et al. Cancer exome analysis reveals a T-cell-dependent mechanism of cancer immunoediting. Nature 2012;482:400-4.

26. Rajasagi M, Shukla SA, Fritsch EF, et al. Systematic identification of personal tumor-specific neoantigens in chronic lymphocytic leukemia. Blood 2014;124:453-62.

27. Birnbaum ME, Mendoza JL, Sethi DK, et al. Deconstructing the peptide-MHC specificity of T cell recognition. Cell 2014;157:1073-87.

28. Su LF, Kidd BA, Han A, Kotzin JJ, et al. Virus-specific CD4(+) memory-phenotype T cells are abundant in unexposed adults. Immunity 2013;38:373-83.

29. Vita R, Zarebski L, Greenbaum JA, et al. The immune epitope database 2.0. Nucleic Acids Res 2010;38:D854-62.

30. van Rooij N, van Buuren MM, Philips D, et al. Tumor exome analysis reveals neoantigen-specific T-cell reactivity in an ipilimumab-responsive melanoma. J Clin Oncol 2013;31:e439-42.

31. Callahan MK, Masters G, Pratilas CA, et al. Paradoxical activation of T cells via augmented ERK signaling mediated by a RAF inhibitor. Cancer Immunol Res 2014;2:70-9.

32. Kitano S, Tsuji T, Liu C, et al. Enhancement of tumor-reactive cytotoxic CD4+ T cell responses after ipilimumab treatment in four advanced melanoma patients. Cancer Immunol Res 2013;1:235-44.

33. Kvistborg P, Philips D, Kelderman S, et al. Anti-CTLA-4 therapy broadens the melanoma-reactive CD8+ T cell response. Sci Transl Med 2014;6:254ra128.

34. Cha E, Klinger M, Hou Y, et al. Improved survival with T cell clonotype stability after anti-CTLA-4 treatment in cancer patients. Sci Transl Med 2014;6:238ra70.
