# Supplementary material for: Contribution of systemic and somatic factors to clinical response and resistance to PD-L1 blockade in urothelial cancer: An exploratory multi-omic analysis
Source: PLoS Med. 2017 May 26;14(5):e1002309. doi: 10.1371/journal.pmed.1002309 (PMC5446110; doi:10.1371/journal.pmed.1002309)
Supplement: S3 Text — (DOCX) [file pmed.1002309.s011.docx]

S1 Supplementary Analyses

Mutation calls, TCR sequencing and RNA sequencing data are available at <http://doi.org/10.5281/zenodo.546110>. All analysis code and all additional data are available at <https://github.com/hammerlab/multi-omic-urothelial-anti-pdl1> for open access by readers.

Mutation loads calculated using the filters from [(10)](https://paperpile.com/c/QI3NxB/O4PR) without Base Quality Score Recalibration (BQSR), or using no filters and no BQSR, demonstrated the highest AUC for outcome ([0.72, 95% CI (0.51, 0.90)](https://github.com/hammerlab/bladder-analyses/blob/master/analyses/notebooks/Different%20Filters%20vs.%20Benefit.ipynb?hyper=without_bqsr_rizvi_filter_auc) and [0.72, 95% CI (0.48, 0.90)](https://github.com/hammerlab/bladder-analyses/blob/master/analyses/notebooks/Different%20Filters%20vs.%20Benefit.ipynb?hyper=without_bqsr_no_filter_auc), respectively, S1 Table). Ultimately, we optimized for precision with respect to variants found on the MSK-IMPACT targeted sequencing panel and chose to use the filters from [(10)](https://paperpile.com/c/QI3NxB/O4PR) with BQSR, which demonstrated a lower AUC for association between mutation load and outcome ([0.65, 95% CI (0.43, 0.85)](https://github.com/hammerlab/bladder-analyses/blob/master/analyses/notebooks/Different%20Filters%20vs.%20Benefit.ipynb?hyper=with_bqsr_rizvi_filter_auc)).

While the main purpose of this study was to improve our understanding of the interactions between somatic alterations, tumor microenvironment and peripheral TCR repertoire, we also note that the present study represents another in the litany of studies exploring biomarkers of response. As such, we recognize that non-invasive and standard-of-care measures represent the ideal biomarkers. Therefore, we examined our cohort with respect to clinical factors known to impact prognosis. When stratified by 5-factor score, patients in the lowest score group (0-1) did not experience a significant improvement in overall survival ([n=26, log-rank p=0.13](https://github.com/hammerlab/bladder-analyses/blob/master/analyses/notebooks/Supp%20Figure%20Extra%20Clinical%20Factors%20Pkyrs%2C%205-factor.ipynb?hyper=fivefactor_os_logrank), S[3A](http://hammerlab.org?figorder=late_deceased_five_factor_plot&group=supp&section=suppall) Fig). As in the overall study [(2)](https://paperpile.com/c/QI3NxB/AkjR), the presence of liver metastases was associated with worse overall survival ([n=29, log-rank p=0.018](https://github.com/hammerlab/bladder-analyses/blob/master/analyses/notebooks/Supp%20Figure%20Extra%20Clinical%20Factors%20Pkyrs%2C%205-factor.ipynb?hyper=liver_os_logrank), S[3B](http://hammerlab.org?figorder=late_deceased_liver_plot&group=supp&section=suppall) Fig).

When we examined only the genes featured in our institution’s next generation sequencing panel, MSK-IMPACT, we did not find an association with DCB ([n=25, Mann-Whitney p=0.42](https://github.com/hammerlab/bladder-analyses/blob/master/analyses/notebooks/Missense%20Filtered%20by%20IMPACT%20Genes.ipynb?hyper=pfs_impact_missense_snv_count_mw)) or OS ([n=25, Mann-Whitney p=0.78](https://github.com/hammerlab/bladder-analyses/blob/master/analyses/notebooks/Missense%20Filtered%20by%20IMPACT%20Genes.ipynb?hyper=os_impact_missense_snv_count_mw)) (S[6A](http://hammerlab.org?figorder=pfs_impact_missense_snv_count_plot&group=supp&section=suppall) Fig, S[6B](http://hammerlab.org?figorder=os_impact_missense_snv_count_plot&group=supp&section=suppall) Fig). There was no association between the APOBEC signature (a vector sum of COSMIC Signatures 2 and 13 [(24,25)](https://paperpile.com/c/QI3NxB/tHzd+SH3r)) ([n=25, Mann-Whitney p=0.23](https://github.com/hammerlab/bladder-analyses/blob/master/analyses/notebooks/Signatures%20-%20APOBEC%2C%20ERCC2%2C%20Smoking.ipynb?hyper=apobec_signature_benefit_mw)) (S[6C](http://hammerlab.org?figorder=apobec_signature_benefit_plot&group=supp&section=suppall) Fig), signature 5 ([n=25, Pearson r=-0.28 p=0.17](https://github.com/hammerlab/bladder-analyses/blob/master/analyses/notebooks/Signatures%20-%20APOBEC%2C%20ERCC2%2C%20Smoking.ipynb?hyper=ercc2_vs_missense_pearsonr)) or the ratio of transitions to transversions and DCB. While there was a significant correlation between the APOBEC Signature and missense SNV count ([n=25, Pearson r=0.40 p=0.048](https://github.com/hammerlab/bladder-analyses/blob/master/analyses/notebooks/Signatures%20-%20APOBEC%2C%20ERCC2%2C%20Smoking.ipynb?hyper=apobec_vs_missense_pearsonr), S[6D](http://hammerlab.org?figorder=apobec_vs_missense_plot&group=supp&section=suppall) Fig), the borderline-significant p-value suggests that this metric merits exploration in a larger dataset. When examining mutations in specific cancer related genes, in addition to variants identified through whole exome sequencing, we also included variants identified using a targeted sequencing panel (MSK-IMPACT, Methods) if available. No tumors exhibited deleterious mutations in *PTEN*; when examined by expression, *PTEN* loss was not associated with DCB; *FGFR3, MYC* and *WNT7B* were also not differentially expressed in patients with DCB versus no DCB, though 4 patients did have *FGFR3* mutations including a frameshift mutation (D785fs) in 9517 (S[6E](http://hammerlab.org?figorder=FGFR3_plot&group=supp&section=suppall) Fig, S[6F](http://hammerlab.org?figorder=MYC_plot&group=supp&section=suppall) Fig). There was only a single tumor (6229) that harbored an *ERCC2* (I80T) mutation.

Given that mutations in the DNA damage response (DDR) pathway could potentially enhance the development of a large number of mutations, we evaluated putatively deleterious DDR mutations based on known or predicted functional impact and found that their occurrence was not associated with DCB ([n=25, Mann-Whitney p=0.20](https://github.com/hammerlab/bladder-analyses/blob/master/analyses/notebooks/DNA%20Damage%20Repair%20with%20Matt%27s%20DDR%20List.ipynb?hyper=num_ddr_possibly_mw), S[6G](http://hammerlab.org?figorder=num_ddr_possibly_plot&group=supp&section=suppall) Fig). It is interesting to note that a single patient with a known germline *BRCA2* mutation experienced a complete response to therapy. A recent report suggested that mutations in *JAK1* or *JAK2*, or dysfunction of beta-2 microglobulin (*B2M*) could lead to immunotherapy resistance (Zaretsky NEJM 2016). There were no mutations in *JAK1, JAK2* or *B2M* identified in this dataset, and there were no significant differences in expression of *JAK1* ([n=26, Mann-Whitney p=0.83](https://github.com/hammerlab/bladder-analyses/blob/master/analyses/notebooks/Kallisto%2BTximport%20JAK1%2CJAK2%2CB2M%2CERCC2%2CFGFR3%2CWNT7B.ipynb?hyper=JAK1_mw)), *JAK2* ([n=26, Mann-Whitney p=0.83](https://github.com/hammerlab/bladder-analyses/blob/master/analyses/notebooks/Kallisto%2BTximport%20JAK1%2CJAK2%2CB2M%2CERCC2%2CFGFR3%2CWNT7B.ipynb?hyper=JAK2_mw)) or *B2M* ([n=26, Mann-Whitney p=0.39](https://github.com/hammerlab/bladder-analyses/blob/master/analyses/notebooks/Kallisto%2BTximport%20JAK1%2CJAK2%2CB2M%2CERCC2%2CFGFR3%2CWNT7B.ipynb?hyper=B2M_mw)) in the DCB versus no DCB groups as measured by PFS.

We examined whether taking expression into account altered the lack of association between mutations, neoantigens, and clinical outcomes. For both missense SNV count and number of predicted neoantigens, we evaluated the association with DCB and with OS greater than 12 months using (a) the total number of variants per megabase, and (b) the number of expressed variants per megabase. In these analyses, the estimated odds ratios for total and expressed variants per megabase were similar to one another irrespective of the metric used ([median OR of 1.38 vs. 1.34 for expressed versus total](https://github.com/hammerlab/bladder-analyses/blob/master/analyses/notebooks/Summarize%20relationship%20between%20mutations%2C%20neoepitopes%20%26%20expression.ipynb?hyper=compare_missense_snv_count_logit_dcb) missense SNV, and [median OR of 1.26 vs. 1.20 for expressed versus total](https://github.com/hammerlab/bladder-analyses/blob/master/analyses/notebooks/Summarize%20relationship%20between%20mutations%2C%20neoepitopes%20%26%20expression.ipynb?hyper=compare_neoantigen_count_logit_dcb) predicted neoantigens when analyzed for association with DCB, with similar results in analysis for association with OS; S[6H](http://hammerlab.org?figorder=coefplot-dcb-pfs&group=supp&section=suppall) Fig, S[6I](http://hammerlab.org?figorder=coefplot-dcb-os&group=supp&section=suppall) Fig). Although we found some degree of differential association with disease progression or mortality in a survival model according to whether the metric was filtered for expressed variants (S[6J](http://hammerlab.org?figorder=univariate-metrics-pfs&group=supp&section=suppall) Fig), the results were not observed in a survival model for mortality (S[6K](http://hammerlab.org?figorder=univariate-metrics-os&group=supp&section=suppall) Fig) and so we considered these findings to be inconclusive. This is corroborated by further analysis looking at whether the rate of expressed to total variants is associated with improved DCB. Given the small size of this cohort, we were not surprised to find inconclusive results ([OR=1.96, 95% CI (0.22, 7.76)](https://github.com/hammerlab/bladder-analyses/blob/master/analyses/notebooks/Summarize%20relationship%20between%20mutations%2C%20neoepitopes%20%26%20expression.ipynb?hyper=summary_missense_expression_ratio_logit_dcb) and [OR=2.34, 95% CI (0.25, 9.34)](https://github.com/hammerlab/bladder-analyses/blob/master/analyses/notebooks/Summarize%20relationship%20between%20mutations%2C%20neoepitopes%20%26%20expression.ipynb?hyper=summary_neoantigen_expression_ratio_logit_dcb) for missense SNV count and predicted neoantigen counts, respectively; S[6L](http://hammerlab.org?figorder=univariate-ratios-pfs&group=supp&section=suppall) Fig, S[6M](http://hammerlab.org?figorder=univariate-ratios-os&group=supp&section=suppall) Fig).

The goal of this analysis is to examine whether taking expression into account altered the lack of association between mutations, neoantigens and clinical outcomes. In total, we considered three metrics that each summarize the mutation load: number of exonic SNVs per megabase, number of missense SNVs per megabase, and number of predicted neoantigens per megabase. Each of these metrics was evaluated twice, once at face value and then again looking only at the number of variants (total SNV, missense SNV, and predicted neoantigens) that are expressed. When each metric was evaluated for univariate association with (a) DCB and (b) OS greater than 12 months in a logistic regression model, the estimated odds ratios for total and expressed variants per megabase were similar to one another irrespective of the metric used (S[6H](http://hammerlab.org?figorder=coefplot-dcb-pfs&group=supp&section=suppall) Fig, S[6I](http://hammerlab.org?figorder=coefplot-dcb-os&group=supp&section=suppall) Fig). In contrast, when the same data are analyzed using a survival model for time to disease progression or mortality (PFS), we see a greater decrease in hazard with each expressed missense SNV, predicted neoantigens or exonic SNV per megabase compared to that observed in similar analysis of the variants alone (S[6J](http://hammerlab.org?figorder=univariate-metrics-pfs&group=supp&section=suppall) Fig). However, these findings are less evident in the model for mortality alone (S[6K](http://hammerlab.org?figorder=univariate-metrics-os&group=supp&section=suppall) Fig). Although these findings, and particularly those suggesting a correlation of increased expression with PFS, are interesting and may be useful for hypothesis generation, the results are inconsistent depending on how the model is specified and so should be interpreted with caution.

In an attempt to corroborate the above findings, we performed an analysis of the expression rate (expressed/total ratio) of missense SNV, predicted neoantigens and exonic SNV variants using both logistic regression and survival analysis. The results were similar to those described above. None of the rates demonstrated an association with DCB or OS greater than 12 months (S[6N](http://hammerlab.org?figorder=coefplot-ratios-dcb-pfs&group=supp&section=suppall) Fig, S[6O](http://hammerlab.org?figorder=coefplot-ratios-dcb-os&group=supp&section=suppall) Fig), however there is evidence that expression rates of missense SNV and predicted neoantigens are correlated with a reduction in hazard in the model for PFS (S[6L](http://hammerlab.org?figorder=univariate-ratios-pfs&group=supp&section=suppall) Fig) but not in the model for OS (S[6M](http://hammerlab.org?figorder=univariate-ratios-os&group=supp&section=suppall) Fig).
